# Tackling Challenges and Exploring Opportunities in Cathode Binder Innovation

**DOI:** 10.1007/s40820-025-01848-4

**Published:** 2025-07-21

**Authors:** Tingrun Lai, Li Wang, Zhibei Liu, Adnan Murad Bhayo, Yude Wang, Xiangming He

**Affiliations:** 1 Yunnan Key Laboratory of Carbon Neutrality and Green Low-Carbon Technologies, School of Materials and Energy, Yunnan University, Kunming, 650091 People’s Republic of China; 2 Institute of Nuclear and New Energy Technology, Tsinghua University, Beijing, 100084 People’s Republic of China; 3 Department of Chemistry, University of Prince Edward Island, 550 University Ave, Charlottetown, PE C1A 4P3 Canada

**Keywords:** Cathode Binder, Lithium-Ion Battery, Performance Optimization, Sustainable Development, Innovative Design

## Abstract

Long-life energy storage batteries are integral to energy storage systems and electric vehicles, with lithium-ion batteries (LIBs) currently being the preferred option for extended usage-life energy storage. To further extend the life span of LIBs, it is essential to intensify investments in battery design, manufacturing processes, and the advancement of ancillary materials. The pursuit of long durability introduces new challenges for battery energy density. The advent of electrode material offers effective support in enhancing the battery's long-duration performance. Often underestimated as part of the cathode composition, the binder plays a pivotal role in the longevity and electrochemical performance of the electrode. Maintaining the mechanical integrity of the electrode through judicious binder design is a fundamental requirement for achieving consistent long-life cycles and high energy density. This paper primarily concentrates on the commonly employed cathode systems in lithium-ion batteries, elucidates the significance of binders for both, discusses the application status, strengths, and weaknesses of novel binders, and ultimately puts forth corresponding optimization strategies. It underscores the critical function of binders in enhancing battery performance and advancing the sustainable development of lithium-ion batteries, aiming to offer fresh insights and perspectives for the design of high-performance LIBs.

## Highlights

- Binders play a crucial role in the lifespan and performance of electrodes, but they are often overlooked. This paper mainly reviews the significance of the role of binders on cathode materials and the optimization strategies.
- Focusing on LiFePO₄ and transition metal oxide cathode systems, this review systematically summarizes performance optimization strategies for novel binders tailored to the respective advantages and limitations of different cathodes.
- The future development trend of cathode binders is analyzed, emphasizing the challenges and opportunities faced by binders in thermal safety and all-solid-state systems.

## Graphical abstract

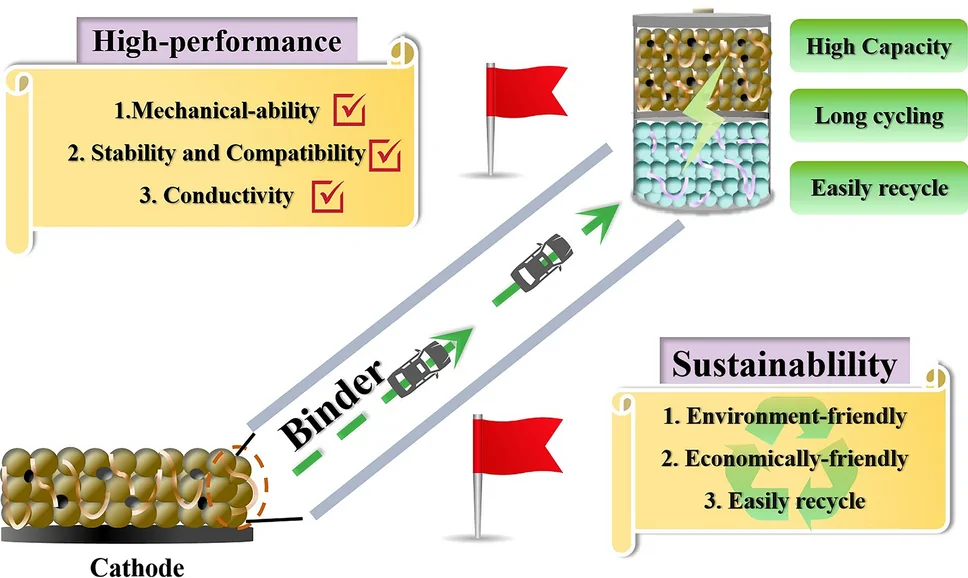

## Introduction

Due to the gradual depletion of traditional fossil fuels and the environmental pollution caused by their production and use, there is a global consensus and trend toward accelerating the transformation of energy structures, improving energy efficiency, and reducing environmental pollution from energy sources [1]. Against this backdrop, in order to drive energy innovation and promote sustainable development, new energy systems are being vigorously developed. Among these, lithium-ion batteries (LIBs) stand out as a highly promising type of new energy storage device, and research focused on achieving their high-performance applications is of significant importance and value [2]. In recent years, to meet the growing energy demand, the focus of researchers has been on how to further enhance the energy density and long-term stable cycling of LIBs' electrochemical performance [3, 4]. Consequently, there has been a surge in interest in improving and optimizing the components of LIBs, including the cathode [5], electrolyte [6], separator, and anode [7–10]. Lithium iron phosphate (LiFePO_4_, LFP) and transition metal oxide cathodes are the most widely used cathode materials by battery manufacturers. Although the energy density of LFP is lower than that of transition metal oxide cathodes, the stability of its olivine structure and the minimal structural contraction and expansion during cycling endow LFP batteries with excellent safety and long-cycle stability, leading to an increasing proportion in the new energy vehicle and energy storage industries [11–13]. Furthermore, with the continuous development of society, the demand for lighter and longer-lasting batteries presents new challenges for the development of LIBs. Under these circumstances, the development and use of electrode materials with higher specific energy have become particularly urgent. A multitude of transition metal oxide cathodes have been developed and applied, to further advance the rapid development of LIBs by increasing the energy density of the cathode materials.

**Fig. 1:**
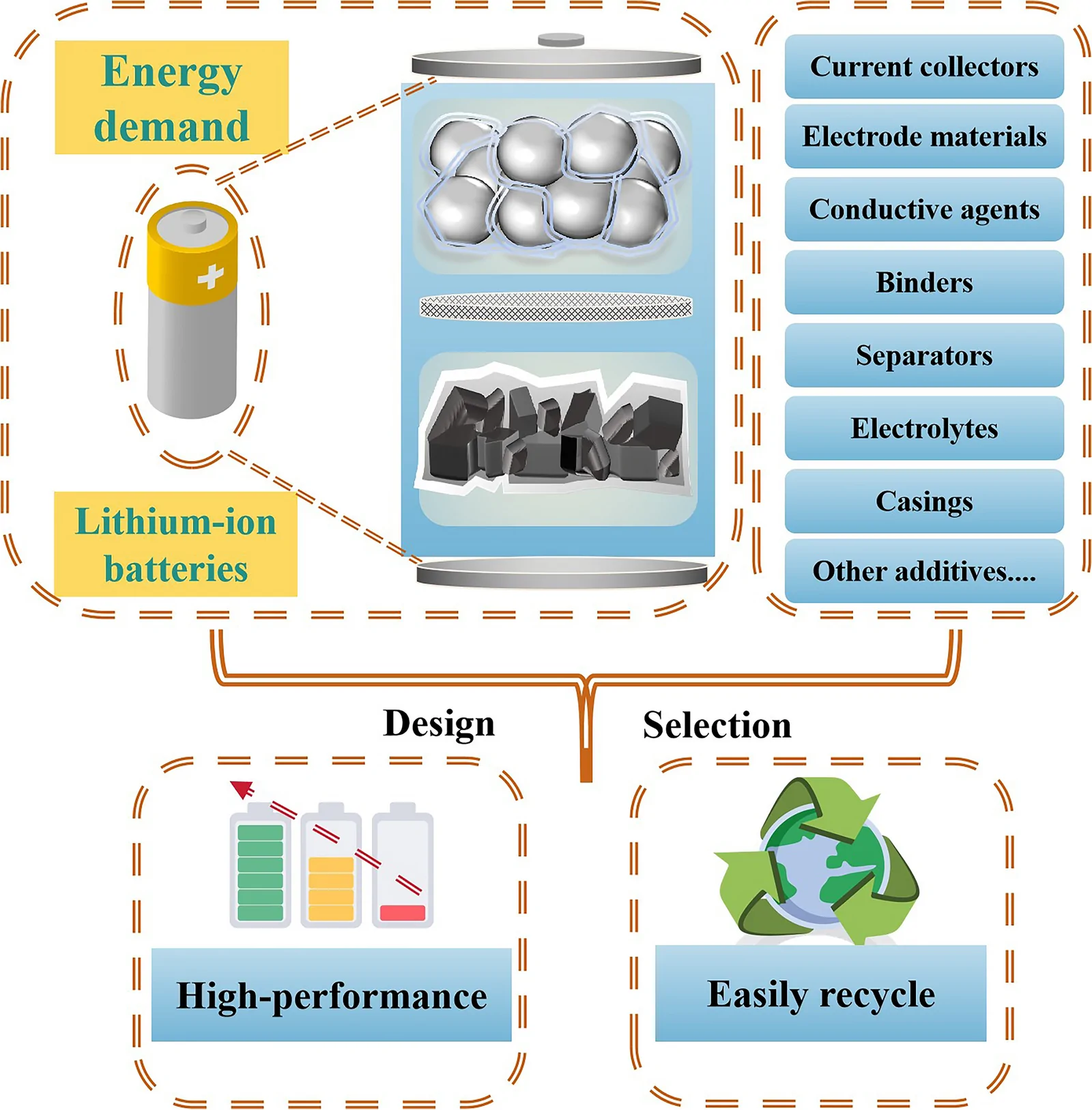
Fossil fuel decline and pollution drive Li-ion battery innovation in energy density, stability, and sustainable recyclable component design. The depletion of fossil fuels and environmental pollution has driven a global trend toward renewable energy and energy efficiency. Lithium-ion batteries (LIBs) are key energy storage devices, and research focuses on enhancing their energy density and cycling stability. LFP and transition metal oxide cathodes are widely used, with LFP known for safety and stability. Transition metal oxide cathodes aim to increase energy density. Other battery components like conductive agents, binders, electrolytes, separators, current collectors, casings, and additives also influence battery performance. Their design and selection are crucial for energy density, power density, cycle life, safety, and cost. Batteries' environmental and economic friendliness in recycling processes are also essential considerations

However, in lithium battery systems, the full play of battery performance not only depends on the intrinsic characteristics of active materials, but also requires the synergistic support of inactive material although they do not participate in electrochemical reactions. They act like an “invisible skeleton” to support the functional architecture of the electrode. Inactive materials represented by binders (conductive agents, separators, electrolytes, additives, current collectors, casings, etc.) transform the theoretical performance of active materials into high cycle life, excellent capacity performance, and reliable safety performance in practical applications through key roles such as maintaining the mechanical stability of electrodes, optimizing the charge transport network, and suppressing interfacial side reactions. In addition, apart from the development requirements of LIB performance, based on the need for sustainable development of lithium-ion batteries, how to achieve environmental and economic friendliness in the battery recycling process has also become a problem that must be carefully considered in battery manufacturing. The selection of binders and the optimization of preparation solvents promote the green transformation of batteries. Therefore, the design of the binder has also become one of the elements that cannot be ignored in battery design (Fig. 1).

As an essential component in the preparation of cathodes, binders play a critical role in firmly adhering the active materials, conductive carbon materials, and current collectors together. Although their content is not high, they are vital for maintaining the structural stability of the cathode, ensuring the rapid and efficient transmission of ions and electrons within the battery, reducing the internal resistance of the electrode, and increasing the energy density of the electrode [14, 15]. The widely used binder is polyvinylidene fluoride (PVDF), which has a broad electrochemical window and remains stable in terms of electrochemical performance between 0 and 5 V, making it less susceptible to oxidation and failure by the cathode [16].

**Fig. 2:**
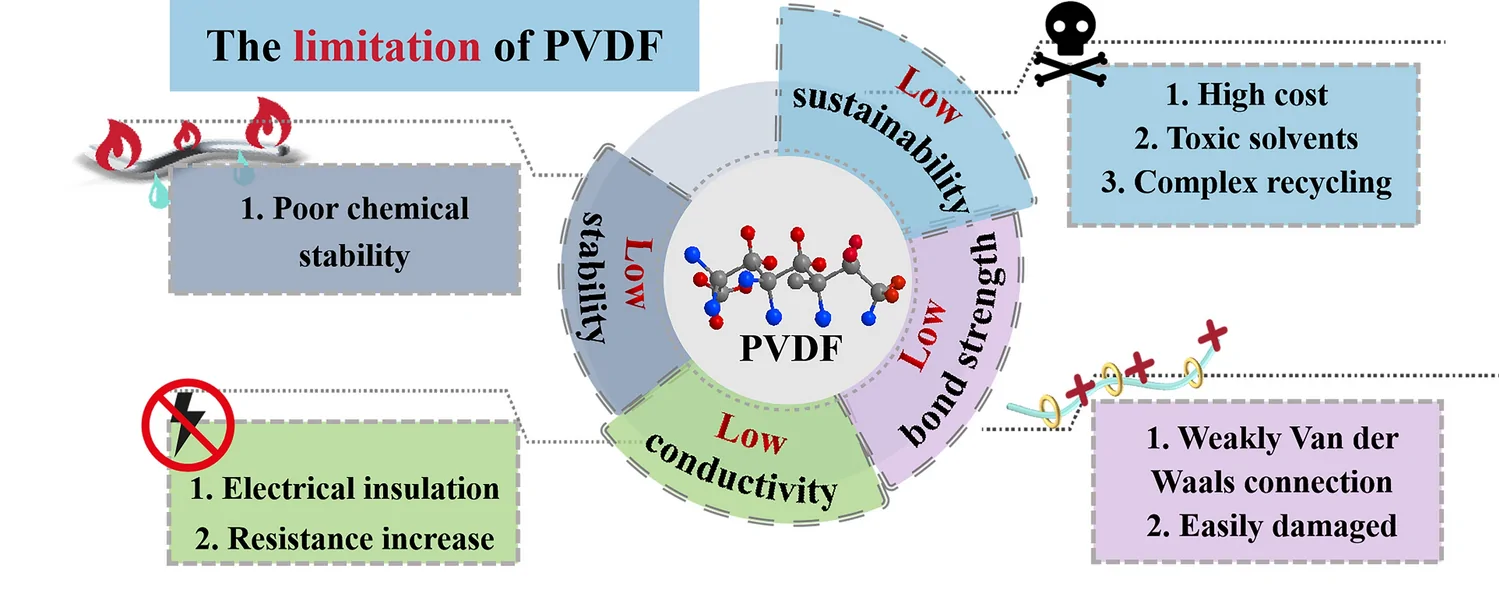
Limitation of traditional cathode binder PVDF. Binders are critical for adhering cathode materials and ensuring battery performance. PVDF is commonly used but has drawbacks like electronic insulation, weak adhesion, and environmental toxicity. Optimizing PVDF or finding alternative binders is necessary for improving battery stability and performance

However, PVDF also has its drawbacks (Fig. 2). It is electronically insulating, which affects the electrochemical behavior of the electrode. Moreover, PVDF primarily generates adhesion through van der Waals forces, and this connection can be easily disrupted when the electrode volume changes during cycling [17], leading to structural damage and capacity loss of the cathode material [18, 19]. Additionally, the use of N-methylpyrrolidone (NMP) as a solvent in the preparation of PVDF is environmentally problematic due to its toxicity, and the cost of recycling is relatively high [20, 21]. The poor thermal stability of PVDF also limits its further application in cathode materials. Because of these defects, it is necessary to reasonably optimize the performance of PVDF as a binder or to seek new alternative cathode binders to replace PVDF.

A qualified cathode binder should simultaneously meet the performance requirements of the cathode and the goals of sustainable development. Therefore, the design of new cathode binders should be reasonably designed through different optimization strategies while meeting the performance requirements of the binder. This paper reveals the shortcomings of traditional binders and summarizes the current research on LFP and transition metal oxide cathode binders, categorizing how they achieve binder performance optimization. The main strategies include: 1) Using non-toxic, environmentally friendly, low-cost, and easily recyclable water-based binders to replace the toxic and environmentally unfriendly traditional PVDF binders. 2) Ensuring the structural integrity of the cathode material during cycling through effective binder design (preventing the shedding of cathode active materials, volumetric expansion causing cracks, and the use of self-healing functionalized binders, etc.). 3) Improving the electrochemical performance of the cathode (including enhancing the effective transmission of ions and electrons within the cathode, reducing the addition of inactive components in the electrode, increasing the cathode energy density, and ensuring uniform distribution of active materials in the electrode). 4) Enhancing the stability of the cathode (including thermal stability and chemical stability against swelling by the electrolyte).

In view of the different characteristics of the LFP and transition metal oxide cathode materials mentioned above, the design optimization focus of the binder also varies. As for LFP, its excellent structural stability has enabled it to develop vigorously in the fields of power and energy storage. In addition, the compatibility of LFP with water-based binders provides a unique advantage for the green development of LFP batteries [22]. By using low-cost and pollution-free water as the solvent, the pollution caused by the need for toxic and volatile organic solvents to dissolve traditional PVDF is avoided [23–25]. However, compared with transition metal oxide cathode materials, LFP cathode materials have obvious disadvantages. Their one-dimensional material structure restricts the ion and electron transfer channels inside the electrode, which is not conducive to the conductivity of the material and ion migration. This leads to a slower reaction rate of Li^+^ within the electrode, resulting in initial capacity loss and poor rate performance of the battery. Therefore, the design of the LFP binder needs to further make up for its capacity disadvantage under the premise of environmental protection and sustainability.

For transition metal oxides, when using more environmentally friendly water-based binders, the sensitivity of transition metal ions (TMs) to water may cause structural changes in the cathode material, thereby affecting battery capacity and cycle performance [26–28]. In addition, the interface instability caused by high pressure and the dissolution of TMs also need to be improved through the design of the binder [29–31].

In addition, with the continuous development and expanding production and application demands of lithium-ion batteries, more requirements are being proposed. Safety issues such as fires and explosions in lithium batteries are continuously exposed during use, restricting their further development [32–35]. In response to these issues, the development of lithium battery systems with superior thermal safety performance has become an urgent need. Traditional PVDF and some water-based organic polymer binders lose their functionality when the temperature reaches a certain point, making it difficult to meet the thermal stability needs at high temperatures [36–38]. How to meet the continuously improving thermal safety needs of batteries through binder optimization strategies is also a challenge that the binder research field will face in the future [39].

Similarly, with the liquid electrolyte system struggling to meet development needs, the emergence of all-solid-state battery systems has also brought new development opportunities for cathode binders [40–42]. Since the all-solid-state system eliminates the electrolyte wetting of the cathode material, there are transmission barriers for ions and electrons in the cathode. Moreover, due to the matching requirements of solid electrolytes, the cathode binder also needs to be adjusted and optimized to meet the performance requirements of batteries in the all-solid-state system. This paper also analyzes and provides development suggestions for these two types of issues, hoping to offer valuable information to the readers.

In summary, as an important additive component in the preparation of cathodes, the binder can be designed to improve the defects of cathode materials and also plays a positive role in the performance of cathodes [43]. However, research on binder modifications has not received widespread attention, and there is a lack of literature summarizing and analyzing the application status and research progress of binders for LFP and transition metal oxide cathodes. This article fills a gap in the literature by offering a comprehensive analysis of the development and application of binders for LFP and transition metal oxide cathodes. It delves into the performance, significance, and current applications of these cathode binders, highlighting their advantages and disadvantages. Drawing from these insights, the article categorizes and summarizes the current strategies for optimizing binder performance. Moreover, it provides forward-looking optimization ideas for cathode binders in line with the evolving needs and development direction of lithium-ion batteries, offering practical suggestions. This work provides valuable design insights for enhancing cathode performance and fostering the development of efficient and sustainable lithium-ion batteries.

## Binders for Cathodes

The binder plays a crucial role in firmly adhering the electrode active material, conductive additives, and other materials to the current collector. It ensures the integrity of the electrode structure during the charging–discharging cycle, preventing the shedding of active material that could compromise battery cycle stability. Although the binder's addition in the preparation of the cathode is not large, it plays a key role in the electrochemical performance of the electrode. Therefore, a systematic exploration and research on the cathode binder is an essential aspect that should not be overlooked for improving the performance of lithium-ion batteries [44–49].

### Role and Functional Requirements of Cathode Binders

**Fig. 3:**
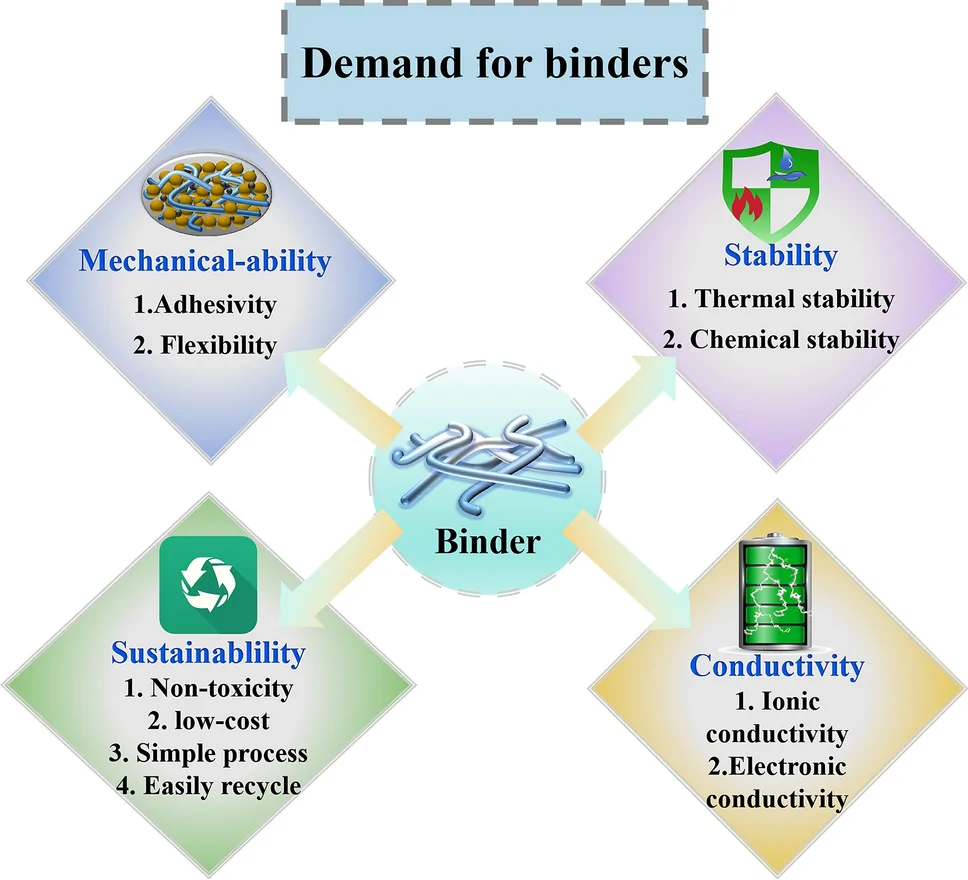
Demand for cathode binders. Binders are crucial for cathode stability and performance, with PVDF being commonly used despite its drawbacks. Optimizing PVDF or finding alternatives is necessary. A qualified binder should meet cathode performance and sustainability goals. Providing with mechanical-ability, stability, sustainability, and conductivity

Cathode electrode binders are essential for electrode structure integrity, mechanical stability, conductivity, electrochemical stability, environmental friendliness, cost-effectiveness, thermal stability, and compatibility with other battery components. They ensure battery performance and safety during charging and discharging cycles (Fig. 3).

**Fig. 4:**
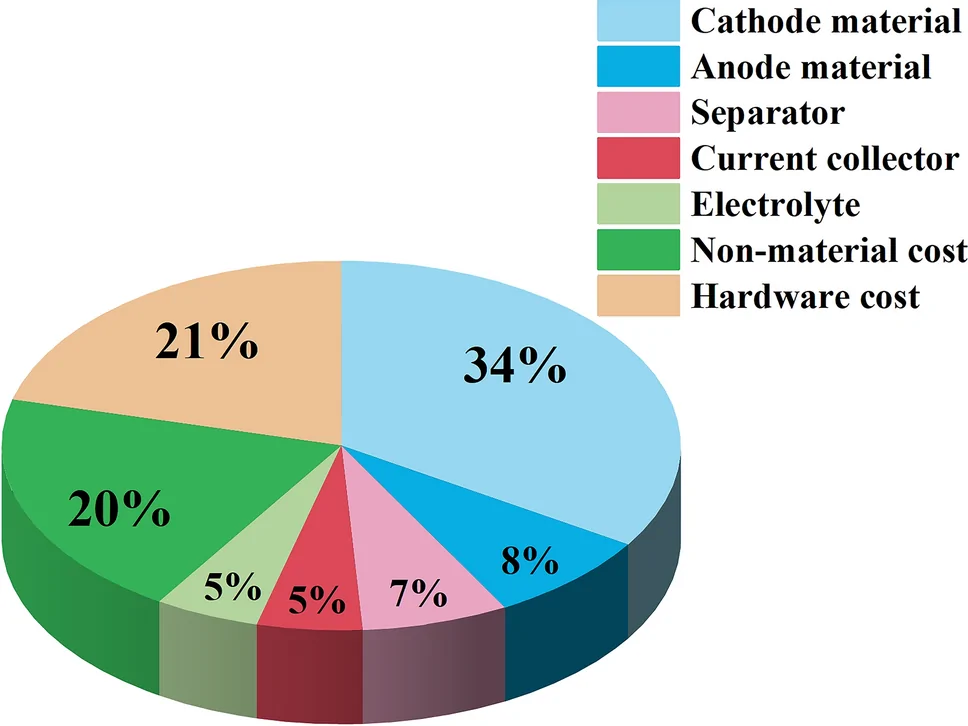
Cost distribution of each component of the battery

For transition metal oxide cathodes with high-energy density advantages, enhancing the transfer efficiency of Li^+^ within the cathode through binder design, shortening the ion–electron transfer paths, is crucial for maintaining and improving the electrochemical performance of these cathodes [50]. However, the introduction of more transition metal elements has intensified the risk of TMs dissolution during cycling, and compared to LFP cathodes, high-energy density cathode materials exhibit more significant volume changes during cycling. To maintain good cycle stability [51], these cathode materials require higher binder adhesion strength, not only to form stable coordination with TMs to prevent dissolution but also to support the cathode structure during larger expansions and contractions, avoiding structural collapse. In terms of battery cost, as shown in Fig. 4, since the cathode cost accounts for a large part of the overall battery production cost [52], the introduction of elements such as Ni and Co further increases the material cost of cathode preparation [53]. Therefore, how to enable the cathode to exert its excellent performance with greater efficiency has become a key issue. In the composition of the cathode, the cost of the binder is relatively low. Therefore, further maximizing the performance advantages of the cathode through the optimization of the binder has become one of the relatively low-cost but highly effective methods [54]. Furthermore, high-energy density cathodes also demand higher binder stability. The high-pressure characteristics of the cathode require a binder with higher resistance to high-pressure oxidation, and the safety concerns associated with high energy density pose new challenges to the thermal stability of the binder.

In summary, the design and selection of cathode binders require a comprehensive consideration of their impact on electrode performance and their role within the entire battery system, aiming to achieve high performance and sustainable development of the battery.

### Efficient Binder Design Strategies

Efficient binder design plays a critical role in electrochemical energy storage and conversion devices such as batteries, especially for electrode materials. Here are some suggestions for binder design based on the characteristics of electrode materials: 1) Electrode Material Type: Design the binder according to the type of electrode material (e.g., metals, metal oxides, conductive polymers, carbon materials, etc.). For example, high-energy density materials such as Li(Ni_x_Mn_y_Co_1−x−y_)O_2_ (NCM) have significant volume changes. The binder needs to have higher elasticity and mechanical strength to buffer the volume changes and prevent electrode cracking. [47]. 2) Electrochemical Stability: The binder should remain stable within the electrode material's potential window without undergoing electrochemical reactions [55]. 3) Mechanical Properties: The binder should have good mechanical strength and flexibility to adapt to the volume changes and mechanical stresses of the electrode material during charging and discharging [56, 57]. 4) Ionic Conductivity: The binder should have a certain level of ionic conductivity to reduce the battery's internal resistance and improve battery performance. 5) Interface Compatibility: The binder should have good compatibility with the electrode material to ensure effective electron transfer and chemical bonding.

Analyzing the design principles of binders from a structural perspective involves the following aspects: 1) Molecular Structure Design: This involves modifying the binder's molecular structure by incorporating specific functional groups to enhance its interaction with the electrode material. 2) Composite Material Design: This involves blending the binder with other materials, such as conductive additives and thickeners, to bolster its overall performance. 3) Cross-linking Network Design: This involves creating a three-dimensional network structure through cross-linking reactions to enhance the binder's mechanical properties and electrochemical stability. 4) Surface Modification: This involves altering the surface of the electrode material to enhance its bonding with the binder.

Another aspect to consider in the design of efficient binders is their functional requirements. This involves assessing the chemical properties, electrochemical behavior, and mechanical performance of the electrode material to optimize the overall performance of the battery. Therefore, efficient binder design should be tailored to the characteristics of the electrode material. Therefore, an excellent cathode binder should have the following basic functional characteristics [58, 59]: 1) Mechanical Properties: This includes adhesion and flexibility to protect the structural integrity of the cathode material during cycling, preventing material shedding or electrode cracks due to volume changes, ensuring battery capacity and cycle stability [60]. 2) Sustainability: Use environmentally friendly binders with non-toxic solvents, low environmental hazard, and lower cost inputs to replace traditional PVDF, avoiding the adverse effects of toxic polar solvents like NMP and reducing the difficulty of subsequent battery recycling. 3) Conductivity: This includes ionic and electronic conductivity to accelerate the rapid and effective conversion of lithium ions in the cathode structure, reduce the electrode's internal resistance, and improve battery electrochemical performance. Additionally, the binder's excellent conductivity can also reduce the addition of conductive carbon in the cathodes, increasing the energy density of the cathode material [61–63]. 4) Stability: This includes thermal and chemical stability, with the binder material having a certain degree of high-temperature structural stability to protect the LFP electrode from temperature increases. Chemical stability requires the binder not to be oxidized by high-voltage cathodes and not to react with the electrolyte, causing swelling and loss of adhesion [64–66].

In summary, efficient binder design for electrodes in energy storage devices requires consideration of material type, electrochemical stability, mechanical properties, ionic conductivity, and interface compatibility. Design principles include molecular structure, composite material, cross-linking network, and surface modification. Functional requirements encompass chemical properties, electrochemical behavior, and mechanical performance. An ideal binder should have strong mechanical properties, sustainability, conductivity, and stability.

### Current State of Application of Binders for Cathodes

The current state of application of cathode binders in lithium-ion batteries is characterized by a variety of materials, each with its own advantages and limitations. The most widely used binder in lithium-ion batteries is PVDF, which offers good mechanical strength, chemical stability, and adhesion to the electrode materials. However, PVDF has limitations such as its sensitivity to high temperatures, which can lead to loss of adhesion and stability, and its use of toxic solvents during manufacturing.

In response to these limitations, researchers have explored alternative binders that offer improved performance in specific areas. Some of these alternatives include: 1) Polyimides: These binders have high thermal stability and can maintain adhesion at higher temperatures compared to PVDF. They are also less toxic and more environmentally friendly. 2) Polyethylene Oxide (PEO) and its derivatives: These binders have good ionic conductivity and can help in improving the overall electrochemical performance of the battery. 3) Polyacrylates: These binders are known for their high flexibility and can accommodate volume changes in the electrode material during cycling. 4) Polyacrylic acids and their salts: These binders have good adhesion and can also form a stable interface with the electrode material. 5) Polyurethanes: These binders offer a good balance of mechanical strength and flexibility, making them suitable for use in various electrode materials. 6) Polypyrrole and its derivatives: These binders can improve the conductivity of the electrode and can also act as a protective layer against electrochemical degradation. 7) Polysiloxanes: These binders have good thermal stability and can also provide a smooth interface with the electrode material.

The choice of binder depends on the specific requirements of the battery application, such as the type of electrode material, the desired operating temperature range, and the desired cycle life. Researchers continue to develop new binders and optimize existing ones to improve the performance of lithium-ion batteries, especially in areas such as energy density, power density, and cycle life.

PVDF, as the most common cathode binder, exhibits good electrochemical stability and is resistant to oxidation by the cathode, making it suitable for use with high-voltage cathodes [38, 67, 68]. This is beneficial for increasing the energy density of lithium-ion batteries. Additionally, PVDF has good wettability with the electrolyte during bonding, which helps to enhance the ionic conductivity within the cathode. However, the disadvantages of PVDF mentioned in the introduction section (low adhesion strength [36, 69], low electrical conductivity [70, 71], and low chemical stability [72, 73]) limit the further improvement of cathode performance. In addition, due to the toxicity and environmental risks of the accompanying solvent NMP, the cost-effectiveness of PVDF usage is greatly reduced [74, 75]. To reduce battery manufacturing costs while meeting bonding requirements, a large number of low-cost, environmentally friendly water-soluble binders have been developed and applied, aiming to replace PVDF in the production of more cost-effective, high-performance LFP cathodes.

The use of water-soluble binders not only improves environmental friendliness and reduces costs but also addresses the issue of weak adhesion strength of PVDF based on intermolecular forces. Many water-soluble organic molecules contain polar functional groups (e.g., -OH, -COOH, -NH_2_, etc.), allowing for bonding through the formation of chemical bonds. This significantly improves the adhesion performance of the binder [58–60]. However, there is still much room for improvement in the application research of water-soluble binders. For example, by optimizing the design, the conductivity of the water-soluble binder can be enhanced to compensate for the poor conductivity caused by the one-dimensional characteristics of LFP materials. This can reduce the use of non-active conductive agents in the electrode, increase battery energy density, and further improve the adhesion performance of the binder by introducing polar functional groups, ensuring the uniform distribution of active materials and enhancing the electrochemical performance of the electrode.

In summary, the conductivity of the binder still needs to be continuously improved in subsequent work to meet the functional requirements of the binder. The third part of this paper categorizes and summarizes the main functional optimization strategies of current binders, aiming to provide reference support for the optimal design of binders.

## Optimization Strategies for High-Performance Binders

The optimization of cathode binders in lithium-ion batteries involves various strategies to enhance performance. These include modifying the molecular structure to improve interaction with electrode materials, combining binders with other materials for enhanced performance, forming a cross-linked network for improved mechanical and electrochemical stability, and surface modification of the electrode material for better bonding. Additionally, optimizing solvents and processes, introducing functional groups, enhancing mechanical properties, improving electrochemical performance, and boosting thermal and chemical stability all contribute to better electrode performance. Interface compatibility is also crucial for effective electron transfer. These strategies collectively aim to boost battery performance, with significant potential for further optimization as demands for higher energy density, power density, and cycle life increase.

### Mechanical Property Enhancement Strategies

When PVDF is used as a cathode binder, it mainly interacts with the cathode material through weak van der Waals forces. This relatively weak interaction leads to poor mechanical properties of the adhesive, such as tensile strength and flexibility, when it undergoes the contraction and expansion changes of the cathode structure. In addition, PVDF is prone to swelling under the erosion of liquid electrolytes, forming gel polymers, which may affect the uniform distribution of electrode materials and the morphology of the electrode. The deterioration of the mechanical properties of the above-mentioned binder may lead to problems such as poor contact, cracks, and even detachment of the positive electrode active material during the cycling process. The degradation of this binder during the cycling process will affect the battery performance. Therefore, it is inevitable to enhance the mechanical properties of the binder through effective strategies to improve the electrochemical performance of lithium batteries, such as capacity retention and long-term cycle stability [76, 77].

To address the inadequate mechanical properties of PVDF, the introduction of hexafluoropropylene (PVDF-HFP) may offer some improvement. There are amorphous regions in PVDF-HFP, which are conducive to the wetting of the electrolyte and ion transport. Meanwhile, part of the crystalline structure is retained to maintain the mechanical stability of the electrode, ensuring the integrity of the electrode structure and the continuity of the conductive network. Hu et al. [62] used the PVDF-HFP binder in the LFP/C electrode and found that it had a relatively good bonding strength. Through mechanical performance testing, it has been found that densified electrodes exhibit different behaviors after being folded three times: PVDF-HFP shows higher adhesion strength, while PVDF electrodes develop cracks or even delamination between the active material and the current collector after folding. Additionally, introducing polymer initiators for cross-linking reactions with PVDF-HFP can form a more stable three-dimensional network structure, further improving its adhesion properties [78]. Designing the binder structure to increase the contact area with the active material may provide a better solution for constructing more stable cathode materials. Generally speaking, the polar functional groups in the binder interact with the active material particles through “point-line” contact, which is not very strong and may deform under high loads. Therefore, changing the contact mode between the adhesive and the active substance may further enhance its adhesion strength. Forming a network structure with nanomaterials to increase the contact area and enabling the binder to interact with the active particles through “surface-point” contact are an effective way to enhance the tensile strength of the binder. Zhang's team [79] used this design idea to interweave carbon nanotubes (CNTs) with non-ionic and nonlinear PVDF to form a framework structure, firmly bonding the active particles through a “surface-point” contact. The rotatable methylene ether bridges in oxidized pullulan (OXP) also provide the binder chain with sufficient flexibility to maintain the stability and integrity of the cathode structure during cycling. Xu et al. [80] optimized the nonpolar and linear binder structure of PVDF through a structural adjustment process, forming a highly organized and microscopically cross-linked binder network. This not only increases the connection strength between the binder and the active material but also provides the binder with higher mechanical strength to better withstand the volume changes of the NCM811 cathode.

However, most modifications are currently based on water-soluble binders due to the demand for environmental friendliness and cost-effectiveness. Many of these water-soluble binders, such as polyacrylic acid (PAA), carboxymethyl cellulose (CMC), carboxymethyl chitosan (C-CTS), polyvinyl alcohol (PVA), polyacrylamide (PAM) [81], and xanthan gum (XG), already contain abundant polar functional groups in their molecular structures, which are beneficial for establishing more stable and stronger connections with the cathode material. For example, C-CTS, a commonly used water-soluble binder, has polar functional groups like -COOH, -OH, and -NH_2_ in its structure, allowing it to form more stable connections with the cathode material through hydrogen bonding or chemical bonding. Researchers have further introduced polar functional groups into C-CTS to enhance its intrinsic adhesion performance. The -CN group, due to the strong electronegativity of the nitrogen atom, can form hydrogen bonds and dipole–dipole interactions, improving the adhesion strength of CN-C-CTS [82], which is found to be 0.047 N cm^−1^, higher than C-CTS's 0.013 N cm^−1^. The enhanced adhesion performance also improves the cycle performance of LFP cathodes.

Huang et al. [83] also used N-cyanoethyl polyethylenimine (CN-PEI) binder instead of PVDF, which effectively maintains the structural integrity of LFP cathodes during the cycling process, resulting in better cycle stability and capacity retention compared to PVDF-bonded cathodes. After 100 cycles at 0.5C, the capacity retention rate can reach 99.6%. Daigle's team [84] believes that PAA, PVA, and other binders have limited structural retention after cycling due to their high T_g_. Therefore, they synthesized a bifunctional emulsion polymer binder. Comparative cross-sectional images of batteries after cycling show that the binder using this bifunctional emulsion polymer exhibits better structural uniformity, with stable contact between the active material and the Al foil current collector, while PVDF shows partial delamination. The specific mechanism of adhesion for this binder has not been fully explained and requires further research.

For transition metal oxide cathodes with significant volume changes during cycling, the design of the binder to effectively withstand the expansion and contraction stresses of the electrode and maintain structural integrity is a key consideration [85]. Wang et al. [86] developed a gel poly(imide-siloxane) binder by in situ cross-linking rigid polyimides with flexible poly(dimethylsiloxane). The rigid segment can effectively withstand the cycling contraction stress of NCM811 electrodes, while the flexible segment provides elastic cushioning to ensure the integrity of the electrode structure. This view can be demonstrated through the peel strength test. The adhesion of the cPIS binder is significantly enhanced to 0.275 N mm^−1^, which is more than twice that of PVDF (0.105 N mm^−1^). Therefore, the interaction force between it and NCM811 particles is stronger, which can improve the mechanical properties of the electrode. It can be found from the SEM of the electrode after cycling NCM811 treated with cPIS binder showed almost no structural damage after 200 cycles.

Additionally, for transition metal oxide cathode materials, the cracks that occur within the cathode particles due to volume changes during cycling can lead to structural damage and the formation of an excessively thick cathode–electrolyte interface (CEI), which reduces electrochemical kinetics at that location and accelerates battery capacity degradation. Therefore, reducing the generation of cracks in this type of cathode not only enhances structural integrity but also prevents the formation of excessively thick CEI interfaces, accelerating the kinetics of electrochemical reactions at the interface.

Currently, the use of a single binder is increasingly unable to meet the multifunctional requirements of the cathode in lithium-ion batteries, and thus, the use of composite binders has become a trend in binder design [43]. Polyacrylate lithium (PAALi) is used as a binder to enhance ionic conductivity due to its additional Li^+^ compensation function. However, PAALi binders are relatively hard, which can lead to the formation of large cracks or even delamination after the electrodes are vacuum dried. To address this issue and ensure that the ionic conductivity and adhesion of the cathode material are both satisfied, thermoplastic polymers such as terpene resin (TS) [87] are often used in conjunction with harder binders to improve the mechanical properties of the binder. The addition of TS improves the flexibility of the binder from 0.12 N cm^−1^ with single PAALi to 0.17 N cm^−1^. Various conductive polymers are also commonly used in conjunction with binders to effectively enhance battery performance. Ye et al. [81] combined a PAM with a stretchable network structure and a conductive polymer, polyaniline (PANI), to form a stable conductive network. The PAM and PANI are cross-linked through N–H hydrogen bonds and chemical bonds between the amide groups and the phenyl rings, providing a stronger bond than typical non-covalent bonds, which helps to maintain the integrity of the cathode structure.

For transition metal oxide cathodes, it is necessary to design the binder to ensure the stability of the cathode material and inhibit the dissolution of transition metal ions (TMs) during cycling. The Kim team proposed a binder design based on amphiphilic brush polymers (BBP) to overcome these challenges. The BBP binder consists of a hydrophobic poly-n-butylenes (PNB) backbone and PAA side chains. The side chains can chelate TMs and enhance the adhesion strength between the binder and the cathode material through hydrogen bonding. At the same time, the PNB backbone's low swelling properties help maintain the structural integrity of the cathode, matching with the NCM811 cathode and maintaining good cycle stability under high loading. Oishi et al. [88] used sulfonated alginate (SO_3_-ALG) to replace PVDF in LiNi_0.5_Mn_1.5_O_4_ (LNMO) cathode materials. During battery charging and discharging cycles, the electrolyte adsorbed on the surface of the LNMO particles reacts with SO_3_-ALG, decomposing products that are captured and deposited to form a passivation protective layer, preventing contact between the electrolyte by-product HF and the LNMO, which can cause Mn^2+^ dissolution. The sulfonic acid groups in SO_3_-ALG also chemically adsorb Mn^2+^, forming stable complexes that can reduce Mn^2+^ dissolution.

**Fig. 5:**
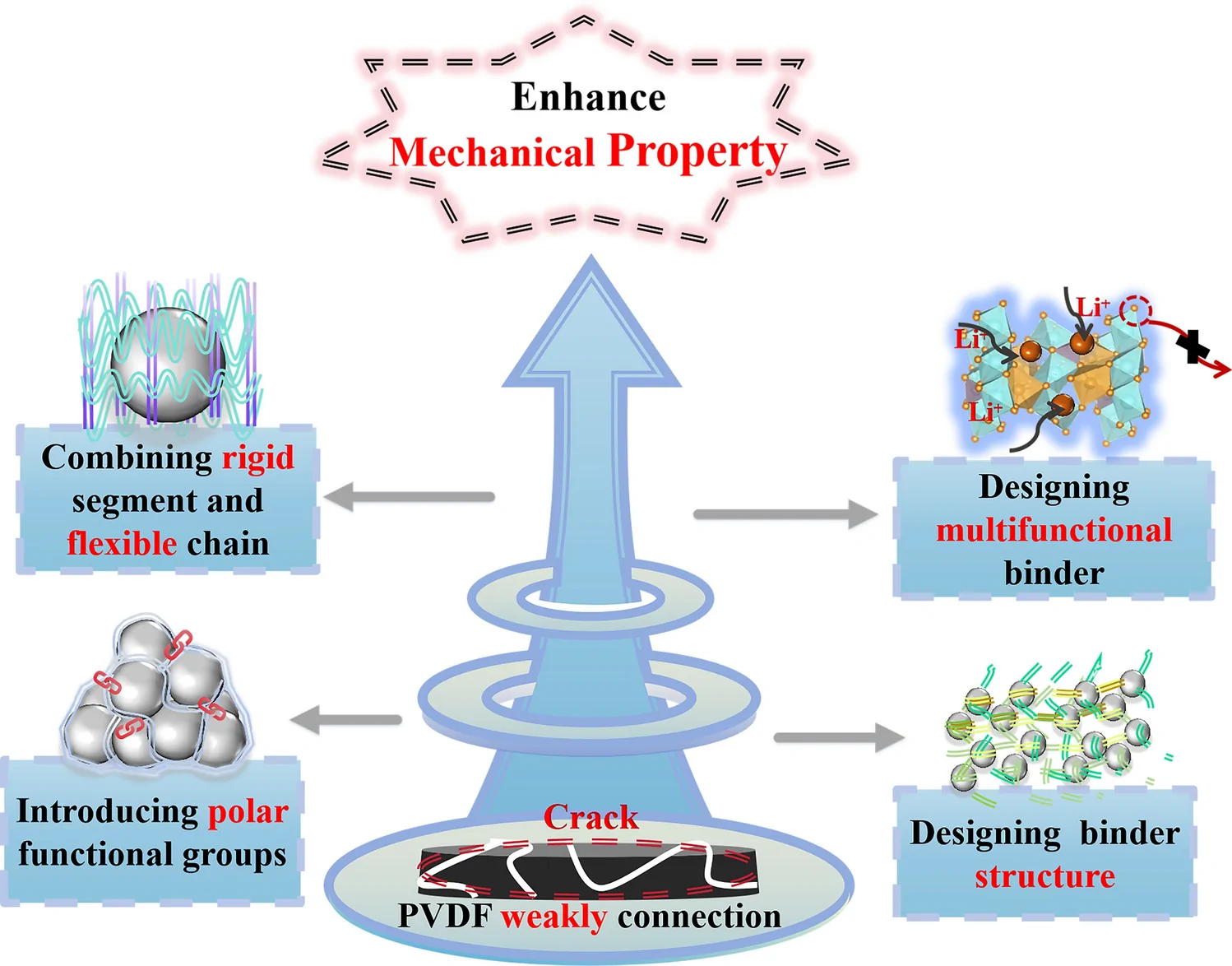
Strategies for enhancing the mechanical properties of binders. PVDF binders have weak mechanical properties and are prone to forming gel polymers, affecting cathode performance. Enhancing mechanical properties involves introducing stronger functional groups and increasing contact area with active material. Transition metal oxide cathodes require binders that can withstand volume changes and prevent TM dissolution

The methods mentioned above mainly involve introducing polar functional groups, increasing the bonding strength of the binder, constructing a stable nanostructured network, and multifunctional composites to enhance the mechanical properties of the binder (Fig. 5). How to choose the appropriate bonding strength of the binder based on the needs of the cathode material to meet the battery performance requirements is also a question that researchers need to consider. In addition to improving the mechanical properties of the binder through various methods, the introduction of polar functional groups can also induce reversible bonding (such as hydrogen bonds, disulfide bonds, and hydrazone bonds) to produce “self-healing” chemical reactions at heating or room temperature. This allows for the spontaneous repair of the binder's structural breakdown caused by volume expansion, stabilizing the cathode structure.

### Strategies for Sustainability

To achieve the sustainable development of lithium-ion batteries and address the environmental and economic issues of binders, the first consideration is to replace the toxic and costly NMP solvent with a non-toxic, environmentally friendly, and low-cost water solvent. Based on this condition, a large number of water-soluble binders have been developed [89, 90]. Prosini et al. [91] used a non-fluorinated dispersible polyvinyl acetate (PVAc) soluble in water as a binder instead of PVDF, reducing production costs and environmental pollution during preparation. The results showed that the PVAc binder exhibited comparable electrochemical performance to the PVDF binder. Chitosan (CTS), a natural organic compound that can be obtained from resources and has no negative impact on the environment, is also used as an excellent alternative to PVDF. Sun et al. [92] used C-CTS as a replacement for PVDF and found that the LFP cathode using C-CTS showed better electrolyte corrosion resistance and cycle performance compared to the PVDF cathode, with a capacity retention rate of 91.8% and 62.1% at 1C and 10C high current rates, respectively, even at 60 °C. In addition to CTS, xanthan gum, a natural extract, has also been used in the development of water-soluble binders for LFP cathodes. He et al. [21] found that the XG-LFP cathode showed better cycle stability and rate performance than the PVDF-LFP cathode, with a higher capacity retention rate at high current rates. Cellulose, through the introduction of carboxyl methyl groups to form water-soluble polymeric CMC, has also attracted the attention of many researchers. Its material cost is about 1–2 EUR kg^−1^, which is significantly lower than the cost of 15–18 EUR kg^−1^ for PVDF, greatly reducing the cost of electrode preparation. Many studies have used CMC to replace PVDF as a binder for LFP cathodes, and Porcher et al. [93] found that CMC can adsorb more solid active materials per unit area than PVDF, with a slightly higher area capacity density. In the CMC binder system, the mass ratio of the conductive agent is also significantly lower than that of PVDF, effectively reducing the proportion of non-active substances in the cathode material. Kim et al. [94] used CMC-LFP cathodes in combination with a non-flammable ionic liquid electrolyte, developing a new lithium-ion battery design strategy that combines safety and environmental performance. In addition to cellulose and natural extract-based water-soluble binders, water-soluble polymer binders such as PAA [16, 95, 96], PVA [97], and styrene-butadiene rubber (SBR) [98] have also been applied in LFP cathodes.

For transition metal oxide cathode materials, water-soluble binders are not commonly mentioned in the literature due to the high sensitivity of TMs to water, which can react with water to produce gases and cause changes in the material structure, affecting battery performance and life [99]. Therefore, the water-soluble binder needs to consider the reaction protection ability of TMs in the binder design. Rolamdi et al. [100] proposed the use of water-soluble polyionic liquids as binders for NCM811 cathode materials. This polymer binder based on poly(diallyldimethylammonium) (PDADMA) and two phosphate anions can dissolve in water and form stable complexes with TMs, preventing their reaction with water and stabilizing the cathode structure, with a cycle capacity retention rate better than PVDF binders. Rao et al. [101] used multifunctional lithium polyacrylic acid (LiPAA) and sodium alginate (Na-Alg) composites for high-voltage LiNi_0.5_Mn_1.5_O_4_ (LNMO) cathode materials. Both can dissolve in aqueous solvents, avoiding the environmental and economic issues associated with NMP solvents. Gan et al. [50] used a water-soluble dextran sulfate-co-polyacrylic lithium (DSS-co-PAALi) to replace PVDF. In addition to eliminating the disadvantages of using NMP, DSS-co-PAALi can also form strong coordination interactions with TM on the surface of the layered oxide through sulfate groups, enhancing the stability of the cathode structure. Not only does it enhance the stability of the cathode structure, but it also effectively inhibits the dissolution of Ni^4+^ and the H2-H3 phase transition of the cathode material. This results in an expanded gap between the Ni 3*d* and O 2*p* energy bands, reducing the activity of oxygen and inhibiting oxygen reduction reactions. The NCM811 cathode after application maintains a capacity retention rate close to 90% after 200 cycles.

Changing the solvent of the binder to reduce environmental pollution and improve economic efficiency is one aspect. However, rational optimization and adjustment of the cathode preparation process to reduce energy consumption and unnecessary cost inputs in the production process are also viable routes to enhance the economic and environmental sustainability of cathode materials. Traditional cathode manufacturing processes using wet methods [102] are relatively cumbersome and include steps such as mixing, coating, drying, NMP recovery, and rolling, with a slow subsequent drying process that consumes a significant amount of energy and time [103, 104]. Therefore, in addition to optimizing the binder material itself, improvements can also be made to the cathode preparation process to reduce the cost and energy loss associated with traditional wet manufacturing processes, thereby enhancing the sustainable development capabilities of lithium-ion batteries.

We understand that in the cathode preparation process using PVDF as the binder, in addition to the cost drawbacks of NMP itself, an NMP solvent recovery step is unavoidably required. Replacing NMP with water significantly reduces the cost input and energy consumption of the recovery step, which is expected to reduce the cost of cathode preparation by a factor of two. However, in the wet process, the drying process cannot be completed in a short time, which also results in a certain amount of energy loss. Therefore, reducing or directly eliminating the use of aqueous solvents will play a key role in reducing the investment and simplifying the process for LFP cathode preparation. Semi-dry processes [105], which aim to optimize the process by significantly reducing solvent usage, are also considered. For these processes, a polymer such as polytetrafluoroethylene (PTFE), known for its strong hydrophobicity and low water absorption, is chosen as the binder. Furthermore, the chain structure of PTFE undergoes disordering at elevated temperatures, leading to volume expansion of the original particles and enhancing electron conduction within the electrode. This can also reduce the addition of non-active materials in the cathode preparation. In this modified process, the binder mass ratio is only 1 wt%, with an energy consumption that is 80% lower than that of the wet process.

With the simplification of the process flow, dry processes [106] have emerged, which simplify a series of operational costs introduced by the use of aqueous solvents, including slurry preparation, coating, and drying. A suitable thermogelling polymer such as propylene carbonate (PPC) is used as the binder in this method, providing both mechanical strength and uniform dispersion in a solvent-free, dry environment. The introduction of this method provides a new direction for achieving more efficient and economical cathode preparation. However, the significant internal stress generated after drying causes cracks in the electrode during the pressing process. In addition, due to the lack of solvent, it is difficult to form a uniform pore distribution before the inactive substance particles, the permeability of the electrolyte is weakened, and ion transport is impaired. These defects will lead to the loss of battery cycle stability and capacity. Moreover, the absence of a solvent as a dispersant poses special requirements for the drying and dispersibility of the binder. In some reports, PTFE [107, 108] has also been used as a binder for dry process techniques.

Embleton et al. [109] addressed the potential problems in current cathode dry processing techniques and introduced a novel solvent-assisted binder (SaB) dry electrode method to meet the sustainable development needs of cathode manufacturing. This method uses a low boiling point and relatively non-toxic ethanol solvent as an aid, with an auxiliary solvent content below 3 wt%, and does not require additional drying steps. The auxiliary solvent can be removed by controlling the pressing temperature. This avoids the issue of potential cracking of the active material in traditional dry processes. The electrodes prepared using the SaB method have a more compact microstructure, higher porosity, and better wettability, which helps to improve the overall performance of the electrodes. After 100 cycles at 1C, the SaB-prepared electrodes achieve a higher capacity retention rate than those prepared using standard dry processes and can operate under high load conditions of 60 mg cm^−2^.

**Fig. 6:**
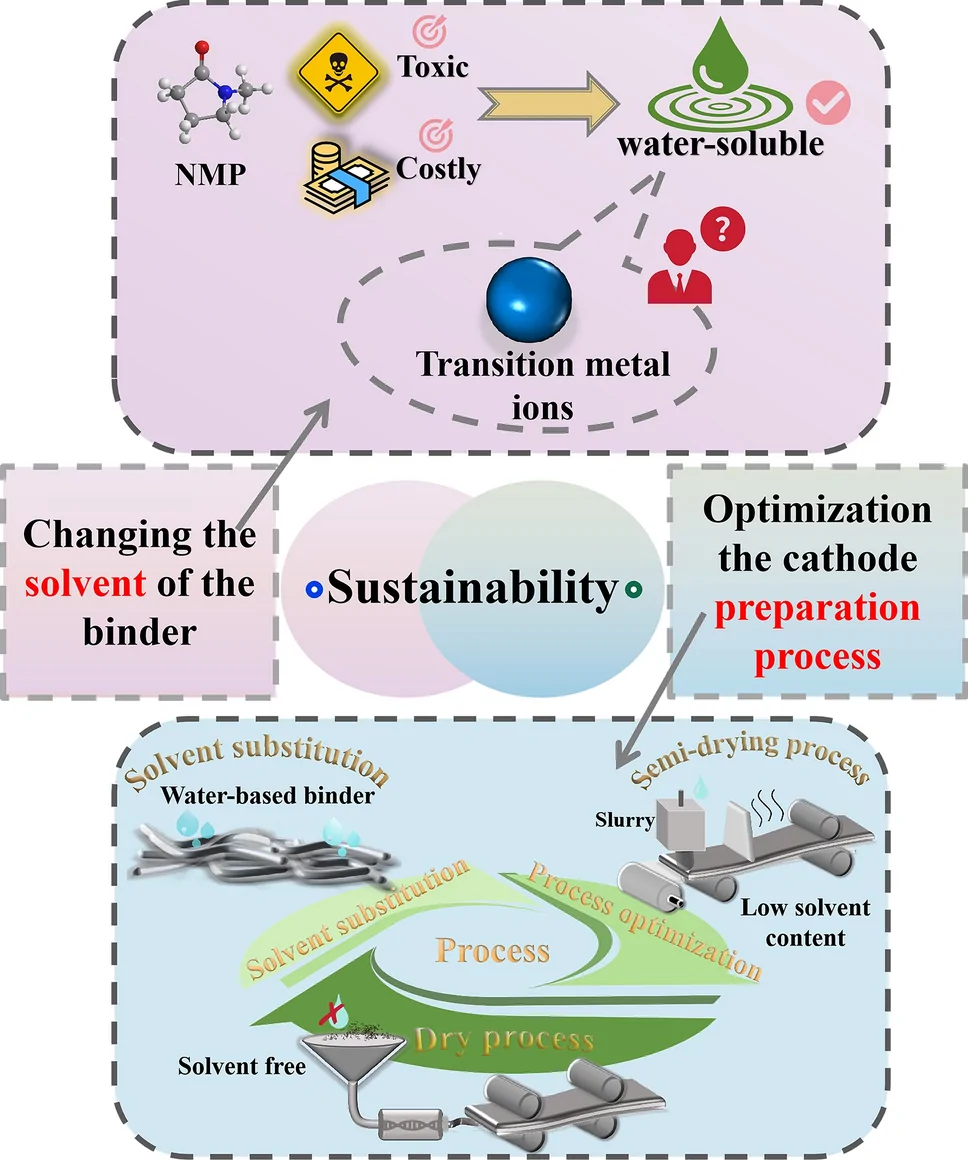
Strategies for enhancing the sustainability of binders. To promote sustainable lithium-ion batteries, water-soluble binders are replacing toxic and costly NMP solvents. However, transition metal oxide cathodes are more sensitive to water, requiring special binder design to prevent TMs reactions. The transition to water-based binders not only reduces environmental and economic burdens but also improves recycling efficiency. Further research is needed to optimize binder performance for both LFP and transition metal oxide cathodes. Changing solvents in cathode binders reduces environmental impact and costs, but optimizing cathode preparation processes can also enhance economic and environmental sustainability. Traditional wet processes are complex and energy-intensive. Semi-dry and dry processes simplify operations and reduce solvent usage. For example, PTFE binders are used in dry processes, and ethanol solvent-assisted methods improve electrode microstructure and performance. Water-based binders are more sustainable but may require further research to maintain electrochemical performance, especially for transition metal oxide cathodes. Continuous process optimization is crucial to ensure lithium-ion batteries meet both environmental and economic goals

The introduction of water-based binders not only provides a more economically and environmentally friendly option for the production and preparation of binders but also makes the collection of active materials from the cathode in the subsequent lithium-ion battery recycling process more convenient and environmentally friendly. Although the introduction of water-based binders provides a feasible alternative to address the environmental and economic issues of cathode binders, from the perspective of overall lithium-ion battery performance, in addition to meeting sustainable requirements such as environmental protection and economy, how to effectively modify the binder material to achieve excellent electrochemical performance of lithium-ion batteries still requires further in-depth research. Particularly for transition metal oxide cathodes, how to minimize the possibility of TMs reacting with water and the harm of side reactions is still a significant challenge. Achieving environmental and sustainable applications of binders without compromising the structure and electrochemical performance of the cathode material is a topic that deserves further consideration (Fig. 6).

In terms of manufacturing processes, while leveraging the energy efficiency advantages of new processes, it is also necessary to refine the preparation conditions and binder types that can efficiently adapt to these processes. This includes continuously addressing technical challenges in new processes to better meet the performance requirements of lithium-ion battery cathodes.

### Conductivity Enhancement Strategies

The capacity of the cathode plays a crucial role in the overall electrochemical performance of lithium-ion batteries. The capacity of the cathode is easily affected by the transfer reaction of Li^+^ ions and electrons within the cathode. As part of the cathode composition, the binder's ionic and electronic conductivity directly affects this transfer reaction. To maximize the cathode capacity, it is essential to ensure that the pathways for electrons and ions within the cathode are continuous and fast. Traditional PVDF binders are electrically insulating and have limited ionic conductivity, which can lead to reduced efficiency of the active material's electrochemical reaction within the electrode, affecting battery performance [110, 111].

Another important factor that affects performance is the distribution state of the cathode active material and the binder. If the various substances in the cathode are not uniformly distributed and agglomerate, the ionic and electronic transport channels will be obstructed, leading to discontinuity, which can also have a negative impact on cathode performance. Therefore, how to improve these deficiencies through rational and effective binder design to enhance the migration and reaction rate of ions and electrons within the cathode is a critical issue that needs to be addressed [57, 112, 113].

For LFP cathodes, restricted by the one-dimensional structure of LFP and the electronic insulating nature of PVDF, the number of mobile charge carriers is greatly reduced, leading to insufficient conductivity. This results in polarization and high internal resistance in the cathode, which is not conducive to maximizing the cathode capacity. For transition metal oxide cathodes, optimizing the internal conductivity of the cathode through binder design is also necessary to maintain and further enhance their rate and capacity advantages [114, 115].

Currently, strategies to enhance the conductivity of binders focus on using binders with better conductivity to replace PVDF. Polyimides (PI) [116] are ideal candidates for LFP cathode binders due to their inherent high ionic conductivity, accelerating the transfer of Li^+^ between active materials and improving the capacity and rate capability of PI-LFP compared to PVDF-LFP. Conductive polymers like polyaniline (PANI) [117] can provide transmission channels for Li^+^ in the cathode, reducing the internal resistance and enhancing the rate performance of PANI-LFP lithium batteries. Ionic liquids with high ionic conductivity are also used to compensate for the deficiency of LFP material's ionic transport. Unlike traditional PVDF, ionic liquids can eliminate part of the Li^+^ diffusion concentration gradient through their high conductivity, allowing Li^+^ to transmit smoothly and quickly to the cathode. An ionic liquid binder synthesized from 1-vinyl-3-ethylimidazolium hexafluorophosphate (VEH) [118] showed a nearly sixfold increase in cathode capacity compared to PVDF at a 5C high rate, demonstrating the crucial contribution of high-conductivity binders to cathode capacity. Rafael et al. [115] prepared an ion–electron conducting binder (MIEC) by mixing conductive polymers PEDOT:PSS and PEDOT:PDADMA-TFSI, which not only provides better electronic contact and ionic transport channels but also reduces the internal resistance and improves battery efficiency, showing better electrochemical performance than traditional binders in both LFP and NCM111 materials.

Oishi's team [88] used sulfonated alginate (SO_3_-ALG) with polar sulfonate groups instead of PVDF. The highly polar sulfonate groups can form stronger interactions with Li^+^ in the electrolyte, promoting the diffusion of Li^+^ within the cathode. The affinity of the binder for the electrolyte also allows for better absorption and distribution, creating more ionic transport channels in the cathode. Liu et al. [119] grafted chlorotrifluoroethylene onto PVDF using atomic transfer radical polymerization as the binder backbone. PAA and block poly(ethylene glycol) methyl ether acrylate (PEGA) are used as side chains for NCM811 cathode materials. PEGA contains hydrophilic polyethylene glycol, which can interact with Li^+^ in the electrolyte to form ion clusters and promote ion mobility. Additionally, the ether bonds and carbonyl groups in PEGA can also form coordination bonds with Li^+^, further reducing the ion migration energy barrier and facilitating ion conduction in the cathode material.

Another strategy to enhance conductivity involves increasing the number of charge carriers in the cathode and shortening the Li^+^ diffusion path through rational binder design. Introducing Li elements into the commonly used water-based binders is a common method of improvement. On the one hand, the introduction of Li^+^ can increase the number of free-moving Li^+^ in the electrode and shorten the ion diffusion path, and on the other hand, the introduction of Li can improve the stripping and insertion efficiency of Li^+^ during charging and discharging, thereby increasing battery capacity. Binders such as CMC-Li [120, 121], PAA-Li [87], SO_3_Li-CS [122], and PSBA-Li [123] have been studied and prepared based on this strategy, and their application has indeed increased the initial charge and discharge capacity of lithium batteries. Introducing negatively charged groups to build connections with Li^+^ through electrostatic interactions is also an effective method to enhance ionic conductivity. Groups such as sulfonic acid [124], cyano [83], carboxyl [61, 125] can all form connections with positively charged Li^+^, reducing polarization during charging and discharging and improving electrode efficiency.

To enhance both ionic and electronic conductivity, the collaborative improvement can be achieved by reducing the content of conductive agents in the cathode [126] to achieve high-density electrodes, increase the energy density of the electrode, and more efficiently achieve chemical reactions of ions and electrons at the cathode, thereby improving the overall electrochemical performance of the battery. The most common approach is to replace traditional non-active conductive agents with conductive polymer (3,4-ethylenedioxythiophene)/poly(4-styrenesulfonate) (PEDOT:PSS) and use it in combination with various water-based binders (such as CMC [127], C-CTS [128], and polyethylene oxide (PEO) [129]), significantly reducing the mass ratio of non-active components in the cathode and increasing the active material content in the cathode from 75%-90% to 92%-99.5% [129]. This results in an excellent binder that combines adhesion, electronic, and ionic conductivity. However, due to PEDOT:PSS being a low-concentration colloidal suspension in water, it can easily lead to uneven distribution of the active material in the cathode, affecting battery capacity.

Dou et al. [130] addressed this issue by co-polymerizing natural polysaccharide alginate with Congo red to form a continuous and uniform ionic-electronic conducting channel within the electrode. This approach improves both conductivity and electrode energy density, with the specific capacity increasing from 108.5 mAh g^−1^ using acetylene black as a conductive agent to 118.8 mAh g^−1^ after 100 cycles.

Graphite oxide (rGO) and other inorganic nanomaterials with excellent conductivity can also be used in combination with organic polymers to prepare cathode binders [131]. An organic/inorganic hybrid conductive binder composed of rGO and an organic compound with a long alkyl chain has been prepared and used, leading to a nearly 30% increase in specific capacity compared to PVDF at a 5C current rate due to the comprehensive improvement in electron–ion transport capabilities.

Improving the ionic and electronic transport capabilities of the cathode material also significantly enhances the rate performance of the battery. However, the design requirements for polymers to achieve ionic and electronic conductivity are different. Aromaticity and high orderliness are beneficial for electronic conductivity, while high dielectric constant and rapid chain migration are required for ionic conductivity. Therefore, it is necessary to design the binder polymer rationally to meet the requirements of both high conductivities. Utilizing electrostatic stabilization to gelate two polymers with opposite charges is an effective method [132]. Unlike simple chain segment composite design, this method produces a binder with more stable chemical properties that is less prone to dissolution in polar electrolytes. The electronic transport capability of the system is improved by three orders of magnitude (from 0.001 to 1 S cm^−1^), and the ionic diffusion coefficient of the cathode is also enhanced. This binder improves the rate performance of the cathode while maintaining stability in carbonate electrolyte systems.

To address the issue of discontinuous or obstructed conduction pathways caused by the uneven distribution of active materials in the cathode, some studies have provided reference solutions [133]. This research primarily focuses on the chemical bonding facilitated by the composite structure of binders, which can effectively interact with the active material particles in the cathode, promoting their uniform dispersion. For instance, the combined use of PAA/PVA forms a stable gel network through hydrogen bonding; the composite binder of polysiloxane and amide salt; and the addition of natural terpene compounds (TX), among others. However, these studies do not provide a detailed explanation of the mechanism by which the binders achieve uniform distribution of active materials, and further research is needed in this area. In addition to the composite binders, structural adjustments to conventional binders can also help alleviate the agglomeration of particles within the cathode.

Shi et al. [134] have in situ polymerized conductive polymers on LFP particles, creating a gel framework with a 3D nanostructure. Due to the high conductivity of the framework polymer and its hierarchical porous structure, the space for continuous electrolyte infiltration is greatly enhanced, facilitating ion conduction. The in situ synthesized binder can uniformly coat the surface of active particles to prevent aggregation. Jeong et al. [135] have effectively improved the surface energy and polarity of the binder by selecting different monomers and adjusting their molar ratios in the binder composition. The resulting poly-norbornene binder ensures the adhesion strength of the active material while also effectively dispersing the active material particles. This constructs a uniform and dense high-Ni layered oxide cathode material structure, achieving excellent battery cycling performance.

**Fig. 7:**
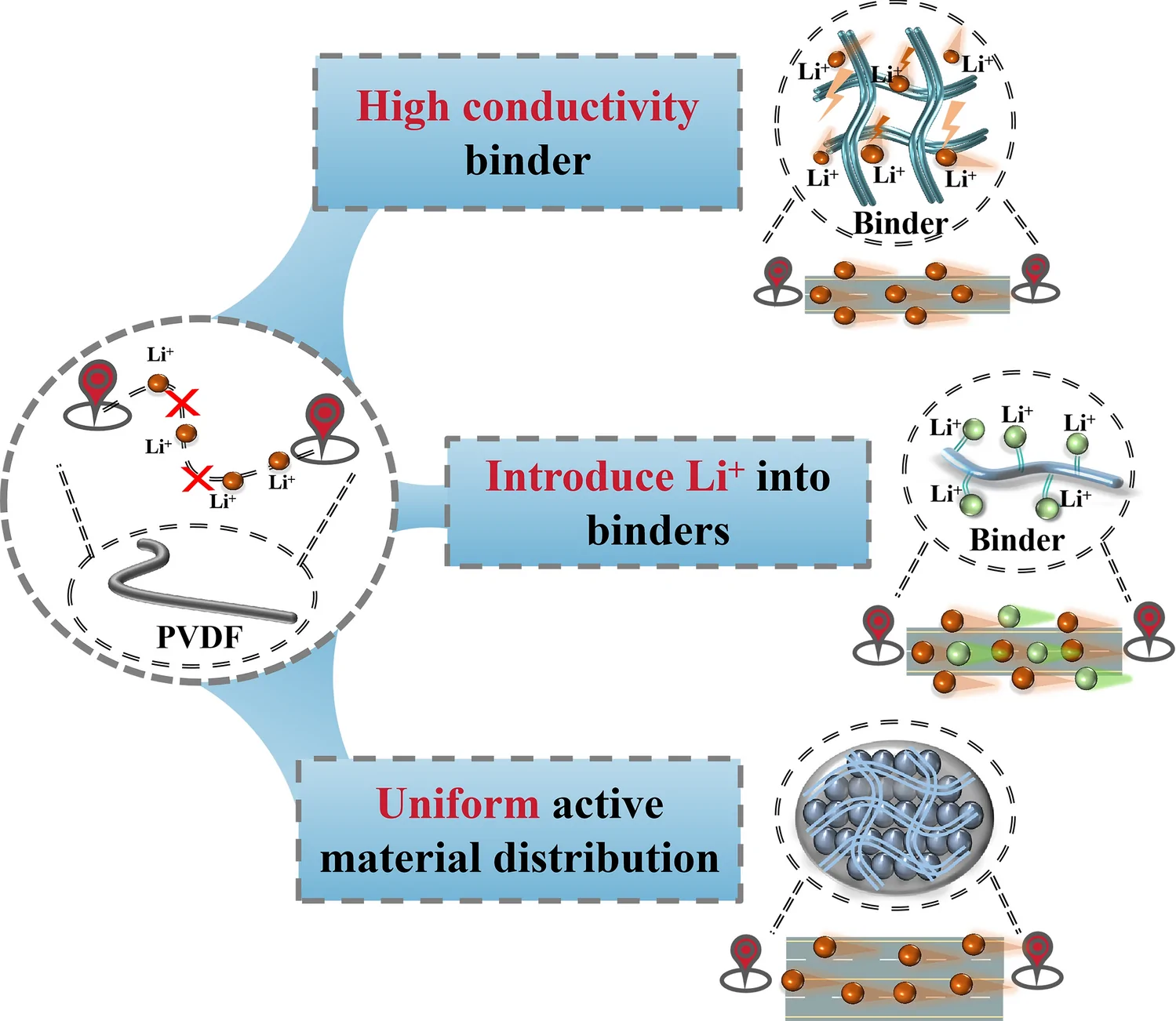
Strategies for enhancing the conductivity of binders. Cathode capacity is critical for lithium-ion battery performance, affected by binder's conductivity and active material distribution. Traditional PVDF binders limit cathode conductivity and uniformity, impacting efficiency and capacity. To enhance cathode conductivity, strategies include improving binder conductivity through materials to improve ion transport and interaction with electrolyte, creating more efficient ionic and electronic pathways in the cathode. Introducing Li^+^ into binders, using negatively charged groups for electrostatic interactions, and reducing non-active components. Structural adjustments like in situ polymerization and surface energy modification also help with uniform active material distribution and electrolyte infiltration. These strategies aim to improve cathode conductivity and maintain electrode integrity, ultimately enhancing battery performance

In summary, the poor conductivity of binders can be largely attributed to the inherent conductivity disadvantage of the material itself and the obstruction of ion and electron transport caused by the uneven distribution and agglomeration of material particles within the cathode (Fig. 7). While various strategies are employed to enhance conductivity, it is also essential to ensure that the new binders are stable and sufficient to maintain the integrity of the cathode structure, ultimately contributing to the overall improvement of the battery's electrochemical performance. Moreover, the analysis of the internal mechanisms behind the enhancement of conductivity should continue to be explored, which will aid in the design of more superior binder systems.

### Enhancement Strategies for Stability

The stability of the cathode is crucial to the overall performance and safety of the battery. Traditional PVDF binders contain fluorine, which is prone to form unsaturated -C = CF- bonds at high temperatures. They are prone to pyrolysis or cross-linking at high temperatures, resulting in a reduction in structural catalysis and bonding strength. Furthermore, the decomposition product HF of LiPF_6_ will attack the unsaturated bonds, causing the main chain of the binder to break and accelerating the degradation of the binder during the cycling process [136, 137]. For high-voltage cathode materials, PVDF is also prone to oxidation and decomposition, accelerating the functional degradation of the binder. The traditional PVDF binder is unable to effectively passivate the interface between the cathode and the electrolyte, forming a stable protective layer to inhibit electrolyte oxidation and the occurrence of side reactions. To sum up, traditional PVDF binder has deficiencies in both thermal and chemical stability, which cannot ensure the efficient application of the cathode.

In light of the stability shortcomings of the PVDF binder, it is crucial to pursue enhancement strategies and explore alternative binders to ensure the effective deployment of lithium-ion batteries. The following strategies can be considered: 1) Developing New Binders with Enhanced Stability: Research and development of new binders that can withstand high temperatures and are less prone to swelling or degradation in the presence of electrolytes. These new binders should have good chemical and thermal stability. 2) Incorporating Stabilizing Additives: Adding stabilizing additives to the PVDF binder or the electrolyte to form a protective layer on the cathode surface, which can prevent direct contact between the cathode and the electrolyte, thereby reducing side reactions. 3) Surface Modification of Cathode Materials: Modifying the surface of cathode materials to improve their compatibility with the binder and enhance their resistance to high temperatures and chemical degradation. 4) Cross-linking Binder Systems: Utilizing cross-linked binder systems that can create a three-dimensional network structure to enhance the mechanical strength and stability of the cathode. 5) Using Inorganic Binders: Exploring the use of inorganic binders, such as polymer-inorganic hybrid materials, which may offer better thermal and chemical stability compared to organic binders like PVDF.

By implementing these strategies, it is possible to improve the stability of the cathode, thereby enhancing the overall performance and safety of lithium-ion batteries.

According to some research findings, LFP cathode batteries exhibit stable performance during cycling tests at room temperature, with no significant capacity fade observed after cycling. However, under elevated temperature conditions, different results are observed. Amine et al. [138] found that when the temperature of LFP cathode batteries is raised to 37 and 55 °C, significant capacity fade occurred after cycling. The researchers attributed this high-temperature capacity fade to the dissolution of Fe ions from the LFP cathode. On the one hand, the dissolution of Fe ions leads to the destruction of the structural stability of the cathode material, which is detrimental to the retention of cathode capacity. On the other hand, once Fe ions are released from the cathode, they can migrate through the electrolyte and deposit on the surface of the anode, participating in the formation of the anode's solid electrolyte interphase (SEI). This process may consume available Li in the anode, leading to further capacity fade.

In response to this issue, more effective binder designs should provide solutions to the problem of metal ion dissolution in LFP cathodes. The carboxyl groups in PAA binder molecules exist mostly as intermolecular dimers at room temperature, exhibiting strong self-association [96], which can provide high binding strength to prevent the exfoliation of active materials in the cathode. At 55 °C, after 100 cycles, the PAA-LFP cathode maintained a capacity retention rate of 97.7%, with no change in the discharge platform potential, significantly outperforming the PVDF-LFP cathode. SEM analysis also revealed that PAA forms a protective structural film on the LFP cathode during cycling, which to some extent inhibits the dissolution of Fe ions from the LFP cathode at high temperatures. Zhang et al. also conducted comparative tests on the swelling behavior of the PAA binder. The results showed that after immersion in a carbonate-based electrolyte for 48 h, the weight gain ratio of the PAA-LFP cathode is less than 5%, while the PVDF-LFP cathode weight gain ratio exceeded 20%. This adsorption and swelling behavior of the electrolyte caused the active particles in the PVDF-LFP cathode to shed, ultimately affecting battery performance.

Similarly, poly(methyl methacrylate) (PMMA) [139] also contains an oxygen-containing functional group ester group, which has a positive effect on maintaining the capacity of LFP cathodes at high temperatures. At 60 °C, the discharge capacity retention of the PMMA-LFP cathode under the same conditions is superior to that of PAA and PVDF, and even higher than the discharge capacity of the PMMA-LFP cathode at 25 °C under the same conditions. This indicates that the stability of the PMMA binder at high temperatures is significantly better than that of PVDF, which is beneficial for the subsequent development and use of thermally stable lithium-ion batteries. Additionally, effectively immobilizing free Fe ions through binder design is an effective strategy to improve the stability of LFP cathodes and prevent battery capacity fade. Ding et al. [140] introduced the ionic liquid (1-butyl-1-methylpyrrolidinium dicyanamide) (PYR14DCA) into the binder, which interacts with Fe ions through the negatively charged free hydroxyl groups produced by the ionic liquid, reducing their subsequent dissolution in the electrolyte and its impact on the anode. Using this method, the capacity retention rate after 1000 cycles at 60 °C is 92%, which is much higher than that of lithium batteries with PVDF binders. For high-voltage cathode materials, the design of a stable binder is not only crucial for preventing the binder itself from oxidation and ensuring adhesion and the integrity of the cathode structure, but it should also form a stable protective structure on the electrode surface. This protection is necessary to avoid unfavorable reactions between the electrode and the electrolyte or by-product gases, thereby reducing the risk of cathode material oxidation. Xu et al. [141] used a multifunctional block polymer (polyetherimide-block-polydimethylsiloxane) as a binder for Ni-rich cathode materials. This binder can inhibit the formation of internal cracks in NCM811 particles and suppress the oxidative decomposition of the electrolyte by regulating the solvation structure of Li^+^. It forms a thin and uniform cathode–electrolyte interphase (CEI) at the cathode–electrolyte interface. The stable interface better protects the integrity of the cathode structure and prevents battery capacity loss.

The Rao team [101] designed a multifunctional aqueous binder, LiPAA-NaAlg, which interacts with the active oxygen atoms on the LNMO surface through carboxylate groups at the cathode–electrolyte interface, forming a dense coating layer. This layer not only inhibits the oxidative decomposition of the electrolyte at the cathode but also enhances the network structure of the binder, further improving its mechanical properties. Chang et al. [73] introduced the concept of a “sacrificial” binder for use in high-voltage LNMO cathodes. They used λ-carrageenan (CRN) as the binder, whose hydroxyl groups can form hydrogen bonds with LNMO to enhance bonding, and the sulfate groups can form ion–dipole interactions with the oxygen atoms on the LNMO surface. Under high voltage, the sulfate groups in CRN can undergo irreversible oxidative decomposition, forming substances like LiSO_x_F (x = 2, 3). These substances not only prevent oxidation reactions between the cathode material and the electrolyte but also effectively address the issue of interface instability in high-voltage cathode materials through a “sacrificial” mechanism, potentially improving Li^+^ conductivity and providing new insights for the design of high-performance lithium batteries. Jin et al. [142] cross-linked PVDF with polyether glycerol ether methacrylate (DPGP) and polyethyleneimine (PEI) through an in situ interweaving method to form a robust three-dimensional network structure. The catechol groups in DPGP can coordinate with TMs in the cathode material, fixing them on the cathode surface and effectively inhibiting the dissolution and migration of TMs. Meanwhile, the abundant ether and amide bonds in DPGP-PEI/PVDF can form hydrogen bonds with F atoms in the electrolyte, reducing electrolyte decomposition and the generation of harmful by-products, thus forming a dense CEI on the cathode surface and reducing side reactions between the cathode material and the electrolyte. In NCM811 batteries, this approach resulted in only 0.02% capacity fade per cycle after 1000 cycles.

To address the issue of TMs dissolution in the positive electrode, the new binder mainly coordinates with TMs in the positive electrode by introducing functional groups (such as carboxyl, hydroxyl, and ester) to form more stable complexes and inhibit its dissolution. In addition, some new types of binders also reduce the dissolution probability of TMs by forming a uniform polymer film on the surface of the positive electrode, physically isolating the erosion of the active material by the electrolyte and some side reaction products.

The strategies mentioned above for enhancing cathode binders are primarily aimed at addressing the deficiencies of traditional PVDF binders and the inherent material defects of the cathodes. By using water as a solvent and introducing aqueous binders to replace the toxic and costly NMP polar solvent, the environmental friendliness and cost-effectiveness of cathode binders are improved. To address the issue of poor adhesion strength due to van der Waals forces in PVDF binders, the introduction of different polar groups enhances the bonding strength between the binder and the components of the cathode, or through rational binder structural design, a more stable cathode structure is constructed to ensure the stability and capacity retention of lithium batteries during cycling.

In response to the less-than-ideal ionic and electronic conductivity caused by structural defects in cathode materials and limitations of PVDF, methods such as incorporating binders with excellent conductive properties or enhancing the uniform distribution level of cathode active particles are employed to improve the conductivity of the cathode binder. This allows for rapid conduction and interaction of ions and electrons within the cathode, reducing internal resistance and polarization phenomena, and increasing the available capacity and energy density of the electrode.

**Fig. 8:**
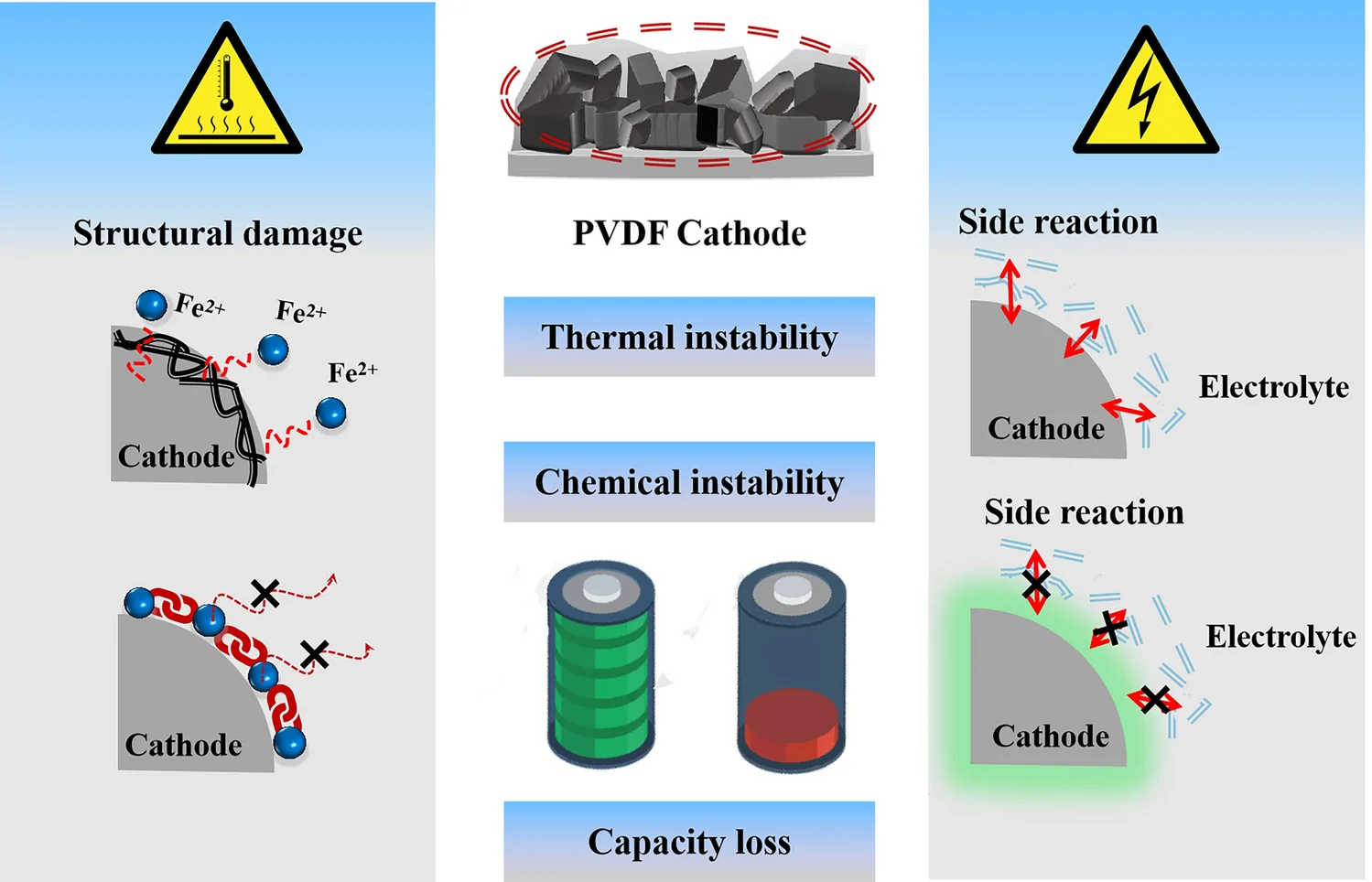
Strategies for enhancing the stability of binders. Traditional cathode binder is unstable at high temperature and high pressure. LFP cathode batteries exhibit stable performance at room temperature but experience capacity fade at elevated temperatures due to Fe ion dissolution. The suitable binder should have high temperature stability, not easy to swell and prevent the dissolution of transition metal ions. Effective binder design for high-voltage cathode materials should prevent oxidation and form a protective interface. Strategies include multifunctional binders, sacrificial binders, and cross-linking to enhance stability, reduce dissolution, and improve conductivity, aiming to meet future battery development needs

To address the issue of insufficient stability of cathode binders, the inhibition of the dissolution of transition metal oxides and the leaching of Fe ions at high temperatures in LFP cathodes is achieved, thereby stabilizing the battery's high-temperature cycling capacity (Fig. 8). However, due to the continuously increasing application demands of lithium-ion batteries, the cathode binder materials that have been studied may not meet the future development needs of lithium batteries.

This review also looks forward to the future application requirements and development directions of cathode binders and offers references and reasonable suggestions, hoping to provide valuable insights for the development of the next generation of LIBs. The suggestions include the need for binders that can withstand higher operating temperatures, maintain mechanical integrity over longer cycles, and improve safety through reduced thermal runaway risks. Additionally, there is an emphasis on the development of binders that can enhance the overall performance of batteries, such as increasing power density, extending cycle life, and reducing cost, all while maintaining or improving the environmental profile of the battery production process.

## Trends in Binders for Cathodes

### Functional Binders with Enhanced Thermal Safety

Functional binders with enhanced thermal safety are a critical area of research in lithium-ion batteries (LIBs), particularly for improving high-temperature performance and preventing thermal runaway [143]. Here are some design principles and strategies for developing functionally enhanced binders with higher thermal safety: 1) High Temperature Resistance: The binder must remain stable within the battery's operating temperature range and be resistant to decomposition, typically implying the selection of polymers with a high thermal decomposition temperature (Td). 2) Thermal Stability: The binder should maintain its chemical and mechanical properties at high temperatures, without softening or degrading. 3) Electrochemical Stability: The binder should not react adversely with the electrolyte or electrode materials, especially under high-temperature conditions. 4) Ionic Conductivity: The binder should possess a certain level of ionic conductivity to aid in ion transport at high temperatures.

Here are some examples of functional binders with potential thermal safety attributes: Polyimides (PIs) are a class of high-temperature-resistant polymers with excellent mechanical properties and thermal stability. Polyphenylene sulfide (PPS) is a polymer with superior heat resistance, capable of maintaining stability in high-temperature environments. Polybenzoxazole (PBO) is a high-performance thermally stable polymer with a very high thermal decomposition temperature and good chemical stability. Polysiloxane exhibits excellent thermal and electrochemical stability, making it suitable for high-temperature battery applications. Ionic liquid binders offer good stability and ionic conductivity at high temperatures and can be incorporated into the binder.

To enhance thermal safety, the design of functional binders can also consider the following factors: Cross-linking can increase the mechanical strength and thermal stability of the binder. Incorporating nanofillers, such as carbon nanotubes or graphene, can improve the binder's thermal conductivity and mechanical properties. Designing multifunctional binders, such as those with both thermal stability and ionic conductivity, can better meet the needs of high-temperature batteries. Developing self-healing binders can automatically repair damage at high temperatures, maintaining the integrity of the battery structure.

Through these strategies, safer and more reliable lithium-ion batteries can be developed, especially for applications in high-temperature environments such as electric vehicles (EVs) and energy storage systems (ESS).

Currently, to address the numerous issues associated with traditional commercial PVDF binders, numerous aqueous binders have been developed and studied. Although these aqueous binders can improve the environmental and economic aspects of the binder preparation process to some extent, they also effectively enhance the adhesion, ionic, and electronic conductivity of the cathode binder, reducing structural damage to the cathode during cycling. However, most aqueous binders are still composed of organic polymer components, and their thermal stability remains insufficient. Traditional PVDF binders have a melting point of about 160 °C. When the internal temperature of the battery reaches 300 °C or higher, the binder will lose its inherent properties, reacting with lithium graphite or the electrolyte to form LiF and unsaturated C = CF- bonds, exacerbating the risk of thermal runaway in lithium batteries and adversely affecting battery safety.

In comparison, some aqueous binders, such as CMC and PAA, which do not contain F atoms in their molecular formula and have a slightly higher thermal stability than PVDF, can improve the thermal safety of lithium batteries to some extent. However, according to some research results, most aqueous binders are still composed of organic polymers, and these organic compounds still lose most of their thermal stability around 400 °C. Seyed et al. [144] found that PAA material exhibits two-step weight loss characteristics in the TGA curve, with weight loss reaching 23% around 300 °C, and when the temperature reaches about 450 °C, PAA undergoes decomposition, causing significant weight loss. This may lead to structural instability or collapse of the cathode material at high temperatures, making it difficult for these aqueous binders to meet the thermal safety requirements of lithium batteries.

Considering the challenges and limitations of traditional organic polymer binders, it is crucial to develop new binder systems with improved thermal stability. The use of water-soluble inorganic binders may provide a new way to improve the thermal stability of cathodes. Compared with organic polymer binders, inorganic binders (such as phosphates and silicates) can usually maintain their structural and performance stability at high temperatures (> 1000 °C) or even under extreme conditions. TG tests show that their weight loss at high temperatures is less than 4%, while commonly used organic binders such as CMC and PAA decompose before 400 °C. In addition, during the water treatment process, inorganic binders hydrolyze to form hydroxyl groups (-OH). After heating and induction, condensation reactions occur, forming covalent bonds with the functional groups on the surface of the electrode material (such as hydroxyl and carboxyl groups). Compared with the hydrogen bonds formed by some organic binders, the bonding strength is greater, thus demonstrating higher adhesion strength and stability.

In addition to their excellent inherent stability at high temperatures, inorganic materials can also absorb the heat released by the cathode material at high temperatures, which is crucial for enhancing the safety of lithium-ion batteries under high-temperature or even overheating conditions. Moreover, inorganic materials inherently possess superior ionic and electronic conductivity compared to organic polymers. These binders can maintain ionic transport capabilities even in the absence of liquid electrolyte infiltration and conductive additives. This is significant for reducing the addition of non-active components in the cathode structure, increasing the carrier transport capacity of the cathode, and improving the cathode's energy density.

In terms of bonding strength, although aqueous binders with polar functional groups such as -OH and -COOH can produce stronger bonds with electrode materials than PVDF due to van der Waals forces, the bonding strength is still lower than that of inorganic binders.

Zhou et al. [145] applied inorganic polymer ammonium polyphosphate (APP) to the cathode of Li–S batteries, and the use of this binder significantly improved the overall thermal safety of the battery. In the case of direct exposure to open flame, the cathode containing APP showed a significant reduction in burning time from 519 s g^−1^ with PVDF as the binder to 289 s g^−1^. Moreover, after the APP cathode is ignited, the ammonia/water vapor released from the decomposition of APP could cross-link to form an insulating polymer layer at the cathode, which prevented the transfer of heat and flammable gases. As a result, the ignited cathode self-extinguished shortly after, preventing further thermal safety accidents. Xu et al. [146] used inorganic sodium silicate instead of PVDF as the cathode binder for Na-ion batteries. The cathode material with this binder showed significantly better thermal stability in TGA tests than the cathode materials bonded with PVDF and CMC. The mass loss is only 7.1% when heated from 30 to 550 °C, compared to 19.0% and 16.2% for PVDF and CMC cathodes, respectively. Additionally, the SMS cathode material released the least heat under high temperatures in DSC tests, which might be due to the thermally stable SMS binder evenly distributed on the cathode material, which is not easily degraded at high temperatures, thus providing high-temperature protection, reducing the oxygen and heat release from the heated cathode, and stabilizing the cathode's performance at high temperatures. The team also tested and compared the bonding strength of SMS through a peel test, and the results showed that SMS had the highest bonding strength of 9 N, while the bonding strengths of CMC and PVDF reached only 4.2 and 1.6 N, respectively. Shivam et al. [147] applied inorganic binders to layered cathode materials, hoping to control the phase transition, oxygen release, and heat generation behavior of layered cathodes at high voltages by adding thermally stable inorganic binders. The exothermic phenomena observed in DSC tests showed that a distinct exothermic peak caused by the phase transition of the material appeared at 316 °C in the PVDF cathode, releasing 428 J g^−1^ of heat. In contrast, the exothermic phenomena of the cathode materials constructed with inorganic binders are significantly suppressed, with the SMS cathode releasing only 38 J g^−1^ of the highest heat value, and the heat released by the cathodes constructed with SPP and STMP is all lower than the PVDF system. These phenomena demonstrate that the addition of inorganic binders can absorb some of the heat released by the cathode phase transition, hinder the accumulation of heat in the battery, and prevent thermal runaway, thereby improving thermal safety.

### All Solid-State System

In batteries containing liquid electrolytes, the immersion of the electrolyte and the addition of conductive carbon provide excellent conditions for ion and electron conduction in the cathode. However, in recent years, due to the demand for battery safety and energy density, lithium-ion batteries with liquid electrolyte systems have gradually been unable to meet future needs. Under these circumstances, solid-state electrolytes have received increasing attention due to their safety advantages such as being less likely to leak or explode. In solid-state systems, however, the cathode is completely dry, which hinders the movement of lithium ions and the conduction of electrons. In this case, using electron-insulating PVDF as a binder further restricts the movement of ions and electrons in the cathode, greatly increasing the internal resistance of the battery. Therefore, in subsequent research and development of cathode binders, it is necessary to develop binder systems that are more adaptable to solid-state environments for all-solid-state lithium-ion batteries [148], ensuring that battery performance is not affected. Rafael et al. [149] believe that in all-solid-state systems, the cathode tends to develop toward denser material structures. The addition of traditional carbon black conductive agents results in a porous cathode in solid-state systems, reducing its energy density. Therefore, they attempted to construct carbon-free cathode binder materials with both ionic and electronic conductivity in all-solid-state systems. They introduced organic mixed ionic-electronic conductors PEDOT:PSS and solid-like ionic liquid organic ionic plastic crystals (OIPCs) to further enhance the material's ionic-electronic conductivity. The overall binder design better adapts to the all-solid-state system, showing a good ion–electron synergy effect at 70 °C, with an electron conductivity reaching 580 S cm^−1^ and an ion conductivity increasing to 3.8 × 10^–5^ S cm^−1^. With this binder, the ion transport activation energy is below 5 kJ.mol^−1^, facilitating rapid Li^+^ transport. Emley et al. [107] conducted a comparative study on the ion conduction differences brought about by different cathode manufacturing processes in all-solid-state battery systems. The team found that the ion conductivity of cathodes prepared by dry processing is nearly 20 times higher than that of wet processing, leading to nearly a 43% increase in electrode specific capacity at the same charge and discharge rates. This result indicates that the dry manufacturing process is of great significance for improving the ion conductivity performance of all-solid-state battery systems. In addition, compared to the wet preparation process, the dry preparation process eliminates the solvent dissolution process and the final solvent evaporation process, greatly reducing the complexity and cost of cathode manufacturing. Beyond the greater demand for ionic conductivity, Lee et al. [150] have addressed the challenges that the widely used wet preparation process for cathodes may face when dealing with solid-state structures. They believe that the biggest problem with the wet preparation process is the need for polar solvents such as NMP to dissolve various materials, which can react with sulfur-based solid-state electrolytes and affect the overall performance of the battery. Therefore, finding binders that can dissolve in low-polarity solvents has become the main issue for their team's research. They selected polybutadiene (PBD) and acrylonitrile butadiene rubber (NBR) as binders, mixed with different mass ratios of acrylonitrile (AN) and tested their performance in solid-state systems. Due to the presence of the -C≡N group, the binder tightly connects with the active material through ionic dipole interactions, overcoming interface contact issues caused by cathode volume changes during cycling, and improving the cycle stability of all-solid-state batteries. Hong et al. [108] also proposed a new perspective on the manufacturing process of traditional cathodes. They believe that the strategy for constructing solid-state battery cathodes should not only replace the electron-insulating PVDF binder but also improve the traditional wet preparation process. To avoid the aforementioned issues with polar solvents, they suggest using a dry preparation process instead of a wet one, eliminating the need for solvents to solve such problems. Finding a more suitable cathode binder that can adapt to the requirements of the dry preparation process while also meeting the performance needs of the binder is particularly important. It is crucial to construct effective ion–electron transport channels while ensuring the structural integrity of the cathode active material. Therefore, the team tried to composite Li^+^ conductive monomers poly(tetrafluoroethylene-co-perfluoro-(3-oxa-4-pentenesulfonic acid)) lithium salt with PTFE as the dry preparation cathode binder to enhance the ion conductivity of the dry preparation binder. However, this preparation method adds a large amount of solid-state electrolyte (25 wt%) to the cathode, which stabilizes the battery's electrochemical performance but does not substantially contribute to increasing the cathode capacity and instead reduces its energy density. Therefore, in subsequent exploration of dry preparation processes the research mentioned above indicates that the current development of all-solid-state systems mainly focuses on solving the internal ionic and electronic conduction within the cathode, as well as optimizing the cathode preparation process. However, there is still a lack of comprehensive research in these areas.

## Summary and Prospects

LFP and transition metal oxides are two types of cathode materials that have been commercialized in LIBs. The different structural characteristics of these materials largely determine their application directions. For the specific functional requirements of batteries, selectively choosing the appropriate cathode material is a key consideration for the rational application of LIBs.

For LFP materials, their inherent cycle stability, safety, and the resource and cost advantages of electrode raw materials give them unique advantages as an important component of lithium-ion batteries. Therefore, the design of binders for LFP batteries can optimize their long-cycle and safety advantages to the greatest extent, meeting the application requirements of such batteries.

For transition metal oxide cathode materials, the demand for high-energy density and high-rate electrochemical performance requires that the binders for these cathodes expand their intrinsic material advantages during the design process. Hence, this review provides a targeted summary and analysis of LFP and transition metal oxide cathode binders, categorizing and introducing their functions. According to the shortcomings of traditional cathode binders and the defects of the cathode materials themselves, the strategies for improving binders are reasonably divided into four parts, which focus on the economic, environmental, sustainability, mechanical properties, electrical conductivity, and stability of the cathode binders, respectively.

Firstly, replacing oil-based PVDF binders with water-soluble binders not only reduces the cost and environmental pollution associated with the recovery of toxic NMP solvents but also creates conditions for the efficient and environmentally friendly recycling of cathodes. It is important to note that water-based binders can easily react with TMs to produce alkaline compounds, which can corrode transition metal oxide cathodes and affect their capacity. Therefore, it is necessary to continuously search for more suitable environmentally friendly water-based binders for these cathode materials.

Additionally, improvements in the cathode preparation process also play a role in reducing cathode cost and energy consumption, and this review provides a corresponding summary and analysis of this content. In terms of mechanical properties, the main strategies for improvement focus on enhancing the bonding strength and flexibility of the cathode binders. This can be achieved by introducing polar groups to enhance bonding strength or by constructing 3D structures and increasing the contact area of the binder, ensuring the structural integrity of the cathode is not damaged during cycling, thereby improving battery stability and capacity retention.

For the enhancement of electrical conductivity, the focus is on improving both lithium-ion and electron conduction capabilities. This can be achieved by introducing conductive polymers, shortening ion diffusion paths, increasing the number of free-moving charge carriers, promoting the uniform distribution of active material particles to create rapid and continuous conduction pathways, reducing internal resistance within the electrode, and enhancing the cathode's conductivity.

Stability improvements concentrate on addressing issues such as the swelling of cathode binders and the prevention of Fe ion dissolution and diffusion from LFP materials after temperature increases, as well as the dissolution of TMs from transition metal oxide cathodes. This ensures the stability of lithium-ion batteries during cycling and the retention of capacity at high temperatures.

**Fig. 9:**
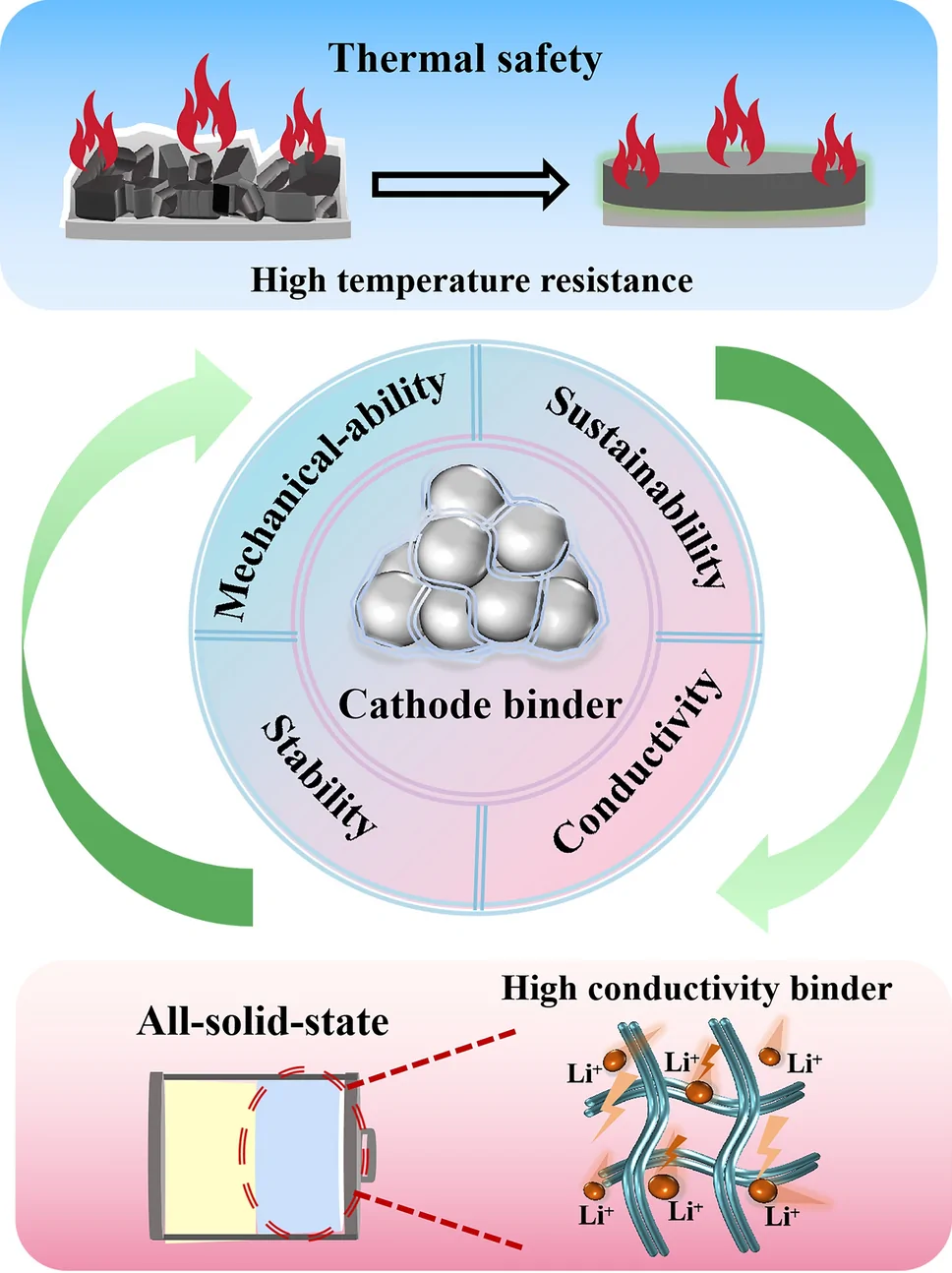
Trends in binders for cathodes. LFP and transition metal oxides are key cathode materials in LIBs, with LFP offering cycle stability and safety, while oxides provide high energy density. Binder design is crucial for optimizing battery performance. Challenges include replacing toxic PVDF with eco-friendly options, enhancing mechanical, stable, and conductive properties. Achieving thermal safety and adapting to solid-state battery demands

The cathode binder faces new challenges as it continues to meet the four major functions mentioned above (Fig. 9). Firstly, the issue of lithium-ion battery safety is becoming increasingly prominent. Most water-soluble polymers are organic, which means that the binder is difficult to maintain stability over long periods at high temperatures. Once the binder fails, it can easily lead to the collapse of the cathode structure, accelerate the thermal failure process within the battery, and potentially cause overall battery failure or thermal safety incidents. Therefore, how to improve the high-temperature resistance of binders is one of the future development challenges. Currently, most research on inorganic binders focuses on the phosphate and silicate systems, with applications mainly concentrated in Li–S batteries or sodium-ion batteries. There is a lack of corresponding research on the use of inorganic binders in lithium-ion batteries, especially in LFP cathodes. Additionally, literature research has revealed that the conductivity of inorganic binders is not outstanding. Some reported phosphate binders still maintain an ionic conductivity in the range of 10^–10^ to 10^–11^ S.cm^−1^ at 60 °C. Subsequent efforts are still needed to find more effective measures to enhance the ionic conductivity of inorganic binders, further expanding their functional application range.

Similarly, under the research background of lithium battery safety, the proposal of solid-state batteries presents another new challenge for cathode binders. The solid-state environment implies a completely dry state without electrolyte immersion, which creates difficulties for the conduction of ions and electrons within the cathode. Therefore, designing cathode binders that are suitable for solid-state environments and ensuring they meet the required ionic conductivity is a challenge that binder design will need to address and solve in the future. Regarding ion conductivity, although there has been some improvement in ion conductivity through current research, it is still difficult to meet the needs of practical applications. Constructing an efficient ionic and electronic conduction network through reasonable binder design in solid-state cathode materials is significant and valuable for improving cathode capacity and the overall electrochemical performance of the battery.

In terms of preparation processes, developing dry processes is a good choice for reducing cathode preparation costs and simplifying the process. However, research on dry processes is not yet mature, especially for applications in all-solid-state systems, which still lacks further exploration. To meet the characteristics of dry preparation processes, commonly explored binders are low-surface-energy polymers that can undergo fibrosis, such as PTFE. However, the chemical stability of these binders needs to be improved. Therefore, to better adapt to solid-state battery systems, it is necessary to continue expanding the types of binders suitable for dry preparation processes. At the same time, it is essential to continuously enhance the ionic and electronic conduction capabilities of binders under solid-state conditions, their bonding performance, and their ability to protect the cathode structure. This will facilitate the advancement of all-solid-state battery technology and its potential for widespread application.

Furthermore, there are also some notable problems in the current development process of cathode binders. Firstly, although water-based binders have resolved the toxicity issue of NMP solvents, they are not entirely safe either. For example, SBR has been listed as a potential carcinogen, so in addition to solvent issues, the toxicity of the binder itself also needs to be carefully observed. The inorganic binder SMS mentioned above also poses potential health hazards to humans, and its acidic aqueous solution may be harmful to aquatic environments. Moreover, in order to further enhance the energy density of the cathode, without affecting the structural stability of the electrode, the mass ratio of the cathode binder should be controlled and reduced as much as possible; binder-free self-supporting electrode may also be a good choice.

In summary, as a component of the cathode that is often overlooked but significant, the rational design of the cathode binder can help lithium-ion batteries achieve more efficient applications. This review aims to provide strong assistance for the subsequent development of cathode binders and promote the performance improvement and research progress of cathode lithium batteries.
